# Ambiguity drives higher-order Pavlovian learning

**DOI:** 10.1371/journal.pcbi.1010410

**Published:** 2022-09-09

**Authors:** Tomislav D. Zbozinek, Omar D. Perez, Toby Wise, Michael Fanselow, Dean Mobbs

**Affiliations:** 1 California Institute of Technology, Humanities and Social Sciences, Pasadena, California, United States of America; 2 University of Santiago, CESS-Santiago, Faculty of Business and Economics, Santiago, Chile; 3 University of Chile, Department of Industrial Engineering, Santiago, Chile; 4 University of California, Los Angeles, Department of Psychology, Los Angeles, California, United States of America; 5 University of California, Los Angeles, Department of Psychiatry & Biobehavioral Sciences, Los Angeles, California, United States of America; 6 University of California, Los Angeles, Staglin Center for Brain and Behavioral Health, Los Angeles, California, United States of America; 7 University of California, Los Angeles, Brain Research Institute, Los Angeles, California, United States of America; 8 California Institute of Technology, Computation and Neural Systems Program, Pasadena, California, United States of America; Seoul National University, REPUBLIC OF KOREA

## Abstract

In the natural world, stimulus-outcome associations are often ambiguous, and most associations are highly complex and situation-dependent. Learning to disambiguate these complex associations to identify which specific outcomes will occur in which situations is critical for survival. Pavlovian occasion setters are stimuli that determine whether other stimuli will result in a specific outcome. Occasion setting is a well-established phenomenon, but very little investigation has been conducted on how occasion setters are disambiguated when they themselves are ambiguous (i.e., when they do not consistently signal whether another stimulus will be reinforced). In two preregistered studies, we investigated the role of higher-order Pavlovian occasion setting in humans. We developed and tested the first computational model predicting direct associative learning, traditional occasion setting (i.e., 1^st^-order occasion setting), and 2^nd^-order occasion setting. This model operationalizes stimulus ambiguity as a mechanism to engage in higher-order Pavlovian learning. Both behavioral and computational modeling results suggest that 2^nd^-order occasion setting was learned, as evidenced by lack and presence of transfer of occasion setting properties when expected and the superior fit of our 2^nd^-order occasion setting model compared to the 1^st^-order occasion setting or direct associations models. These results provide a controlled investigation into highly complex associative learning and may ultimately lead to improvements in the treatment of Pavlovian-based mental health disorders (e.g., anxiety disorders, substance use).

## Author summary

In everyday life, we learn to associate various situations with various outcomes. For example, perhaps a specific person usually receives praise (outcome) when giving a public speech (situation), but if they give a speech after a particularly charismatic speaker (situational factor), their speech might receive less praise than usual. In our report, we conducted two experiments investigating how people learn about highly complex associations. By highly complex, we mean that whether stimulus 1 (S1) leads to outcome 1 (O1) depends on whether stimulus 2 (S2) was present or absent, and S2’s effect on the S1/O1 association may further depend on whether stimulus 3 (S3) was present. Previous work has shown that S2 will modulate S1’s association with O1 using two hierarchies of stimulus value, where S2 is a higher-order stimulus that affects the lower-order S1/O1 association. We hypothesized a third hierarchical value exists if S3 affects how S2 modulates the S1/OS1 association. We created formulas predicting three hierarchical levels of learning, and we tested our three-level hypothesis and formulas in two experiments, finding strong support for both. Through the experiments, we show that humans have the ability to learn highly complex associations, which suggests we may do so in everyday life. Our formulas provide insight into the computations our brains may engage in while learning. This has relevance for increasing our understanding of associative learning (which has been largely limited to a two-level hierarchy to date), as well as mental health disorders related to associative learning (e.g., anxiety, substance use).

## Introduction

Real-life learning is filled with associations that range from being very simple to very complex. For example, most people have learned that when a streetlight turns green, cars will start driving. This association is simple and consistent with very few exceptions. As a more complicated example, imagine a person who is about to give a public speech. Will the speech go well? It depends. What is the speech topic? How competent is the speaker on that topic? Who is the audience? How much did the speaker prepare? How well did the speaker sleep the night before? There are many situational factors that influence whether a speech will go well, and there are many life examples like this in which it is unclear or ambiguous as to whether a given stimulus/situation will result in a specific outcome. To date, most research has focused on simple or moderately complex associative learning, but very little work has been conducted on highly complex associative learning. By investigating highly complex and ambiguous associations, we take an important step towards understanding how humans learn about real-life associations, which are often highly complex.

The most prominent experimental paradigm for learning stimulus-outcome associations is Pavlovian conditioning, in which the individual learns associations between conditional stimuli (CSs) and their outcomes (i.e., unconditional stimuli; USs). The CS+ is a CS that is paired with the US, whereas the CS- is paired with the absence of the US. In many experiments, the CSs tend to be *unambiguous* stimuli–meaning, they always (or almost always) predict the presence or absence of the US. However, in the real world, it is rare for CSs to be truly unambiguous. For example, whether a speech (CS) will result in a specific outcome–such as praise, rejection, or something in between–is usually ambiguous and depends on many situational factors.

One of the primary Pavlovian experimental designs investigating ambiguous CS/US associations is occasion setting [1–8], in which one stimulus (i.e., the occasion setter) indicates whether an ambiguous CS will result in the US. For example, perhaps a specific child is usually talkative during dinner, but the parent is interested in figuring out which situations lead the child to be quiet during dinner. From a Pavlovian perspective, the parent is interested in learning which stimuli, situational factors, or contexts determine whether the child (CS) will talk (US) or be silent (no US) during dinner. In this case, perhaps when the child spends time with a particular friend in the afternoon, the child is quiet during dinner. Theoretically, spending time with the friend (occasion setter) would signal that the child (CS) will be quiet during dinner (no US), but not spending time with the friend would signal that the child (CS) will be talkative during dinner (US).

There are two types of occasion setters: positive and negative. Positive occasion setters signal that the CS will predict the US (i.e., the CS predicts the US *only if* the positive occasion setter was presented). The public speech example would be positive occasion setting if the person believes their speech (CS) will result in rejection (US) only if they give a speech after a particularly charismatic and engaging speaker (positive occasion setter). Negative occasion setters signal that the CS will not predict the US (i.e., the CS predicts the US *unless* the negative occasion setter was presented). The friend/child example above is an example of negative occasion setting, where the friend (negative occasion setter) signals that the child (CS) will be quiet during dinner (no US).

**Fig 1:**
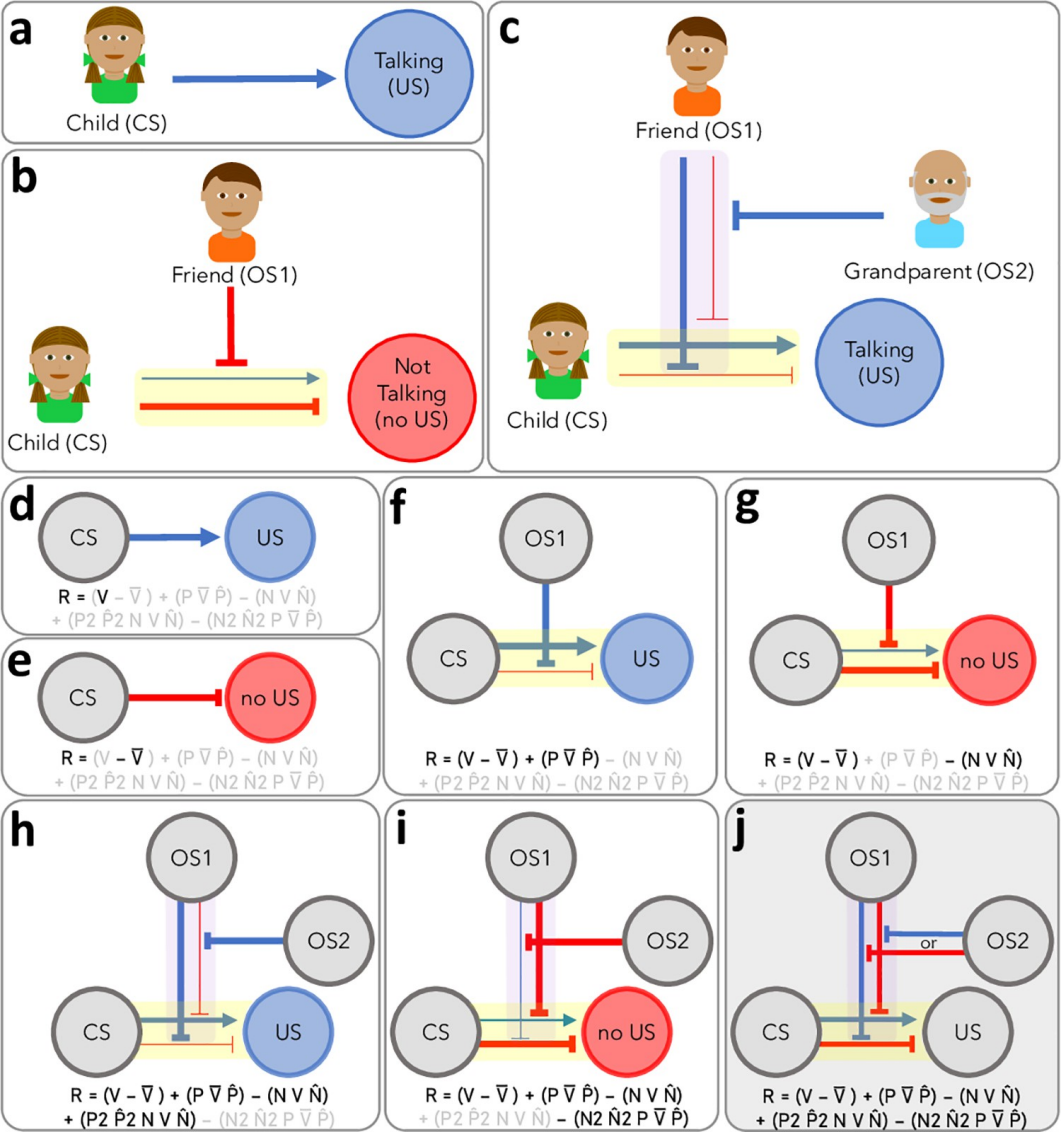
Hierarchical Model of 2^nd^-Order Occasion Setting. Using example from main text of child, friend, and grandparent: a) direct associative learning, b) 1^st^-order occasion setting, and c) 2^nd^-order occasion setting. Panels d-j display: d) direct excitation, e) direct inhibition, f) 1^st^-order positive occasion setting, g) 1^st^-order negative occasion setting, h) 2^nd^-order positive occasion setting, i) 2^nd^-order negative occasion setting, and j) the total model with all associations. Our mathematical model is presented at the bottoms of panels d-j, where black/bold variables are active (i.e., values greater than 0), and gray variables are inactive (i.e., values are 0). See Table 3 for details on formulas. In the figure, circles are stimuli: unconditional stimulus (US), conditional stimulus (CS), 1^st^-order occasion setter (OS1), and 2nd-order occasion setter (OS2). Blue arrows indicate direct excitation; blue line segments indicate positive occasion setting; red line segments indicate direct inhibition or negative occasion setting; yellow glow indicates CS ambiguity; purple glow indicates OS1 ambiguity; blue USs indicate US delivery; and red USs indicate US omission. While we suggest that stimulus ambiguity is a dimensional, learned property, we present it as present/absent in the figure for simplicity. Thick arrows/lines indicate activated pathways; thin arrows/lines indicate deactivated pathways. Stimulus ambiguity is required for higher-order associative learning: 1^st^-order occasion setting is learned only if the CS is ambiguous and has been trained with an OS1; 2^nd^-order occasion setting is only learned if the CS and OS1 are ambiguous and if the CS has been trained with an OS2. CSs have a direct predictive relationship with the US. If the CS is ambiguous (i.e., sometimes predicts the US, sometimes predicts absence of the US), then attention is broadened to other stimuli or contextual factors (i.e., to the OS1); if a stimulus that disambiguates CS reinforcement is identified and is less salient than the CS, it becomes an OS1. The OS1 modulates the CS/US association. If OS1 consistently excites the CS/US association, then OS1 is a positive OS1; if OS1 consistently inhibits the CS/US association, then OS1 is a negative OS1. If OS1 sometimes excites and sometimes inhibits the CS/US association (i.e., OS1 is ambiguous), then attention is broadened to other stimuli or contextual factors (i.e., OS2) that disambiguate how the OS1 affects the CS/US association. If a stimulus disambiguates the effect of OS1 on the CS/US association and is presumably less salient than the OS1, 2^nd^-order occasion setting is learned. If the OS2 consistently disables the OS1’s 1^st^-order positive occasion setting ability, the OS2 is a 2^nd^-order negative occasion setter. If OS2 consistently disables OS1’s 1^st^-order negative occasion setting ability, then OS2 is a 2^nd^-order positive occasion setter. Additionally, each hierarchical level (direct associations, 1^st^-order occasion setting, 2^nd^-order occasion setting) and excitatory/inhibitory directions are orthogonal–meaning, a given stimulus can be any combination of an excitatory or inhibitory CS, OS1, or OS2 (e.g., a given stimulus can simultaneously be a CS+, negative OS1, and positive OS2).

Occasion setting is thought to operate via modulation [1,7], where the occasion setter affects the CS/US association. The modulation account posits that stimuli are arranged hierarchically, where higher-order learning (i.e., occasion setting) affects lower-order learning (i.e., direct associations: learning that a CS directly predicts the presence or absence of the US). Many studies have been conducted on traditional occasion setting, described above ([1,2,7–18]; hereafter referred to as 1^st^-order occasion setting), but there is very limited research on 2^nd^-order occasion setting, including studies investigating it directly [19] or indirectly [9,16,20,21]. We define 2^nd^-order occasion setters as stimuli that determine how *ambiguous* 1^st^-order occasion setters will affect the CS/US association. Using our example above, the child (CS) is ordinarily talkative during dinner (US) unless they see their friend that day (1^st^-order negative occasion setter). Converting this to a 2^nd^-order occasion setting example, perhaps the friend only *sometimes* causes the child to be quiet during dinner, making the friend an ambiguous 1^st^-order occasion setter. A 2^nd^-order occasion setter would determine whether the friend (1^st^-order occasion setter) will cause the child (CS) to be quiet during dinner (no US). Perhaps the child’s grandparent (2^nd^-order positive occasion setter) gives the child good advice regarding the importance of family time that causes the child (CS) to talk during dinner (US)–even after the child sees their friend in the afternoon (1^st^-order occasion setter). This type of example more closely resembles the complexities of real-life associative learning, where stimulus-outcome associations are usually ambiguous and dependent on situational factors. See Fig 1 for our model of 2^nd^-order occasion setting, in which higher-order learning can only occur when lower-order stimuli are ambiguous (i.e., when lower-order stimuli are sometimes followed by the US).

### The Present report

While 2^nd^-order occasion setting is a theoretically plausible learning process, there are no clear demonstrations of it. Additionally, there are no formal models that predict 2^nd^-order occasion setting–perhaps because 2^nd^-order occasion setting has not been explicitly demonstrated. Thus, there were two goals in the present experiments: 1) determine whether 2^nd^-order occasion setting can be learned (as a model of highly complex associative learning), and 2) evaluate our computational model of 2^nd^-order occasion setting to see if it is a more accurate predictor of learning than simpler models (i.e., 1^st^-order occasion setting or direct learning). To this end, we conducted two mirror-image experiments: a 2^nd^-order negative occasion setting experiment (Experiment 1) and a 2^nd^-order positive occasion setting experiment (Experiment 2). To address the first goal, we trained multiple stimuli across discriminations intended to produce learning across three hierarchical levels: direct learning, 1^st^-order occasion setting, and 2^nd^-order occasion setting. We conducted specific tests (i.e., transfer tests) to test whether 1^st^-order and 2^nd^-order occasion setting were indeed learned, in which we would expect the occasion setters to transfer their effects to lower-order stimuli who underwent similar occasion setting training but not to stimuli that did not undergo occasion setting training. As an additional assessment, we predicted that each hierarchical level would be orthogonal–meaning, a stimulus could signal outcomes in each of the three levels (e.g., Stimulus A would be a CS+ when presented alone, a 1^st^-order positive occasion setter when preceding a CS, and a 2^nd^-order negative occasion setter when preceding a 1^st^-order occasion setter and CS; [1,2,7,15,22]). To address the second goal, we conducted Bayesian hierarchical modeling to evaluate model fit using our novel 2^nd^-order occasion setting model and contrasted its predictions with our simplified 1^st^-order occasion setting model and our even more simplified direct associations model. If the data fit our 2^nd^-order occasion setting model better than the 1^st^-order occasion setting model and direct associations model, this would provide computational support that 2^nd^-order occasion setting was indeed learned. For a list of all specific hypotheses and analyses details, please see our pre-registrations (Experiment 1: https://osf.io/n2c6v, Experiment 2: https://osf.io/hxcfs).

**Fig 2:**
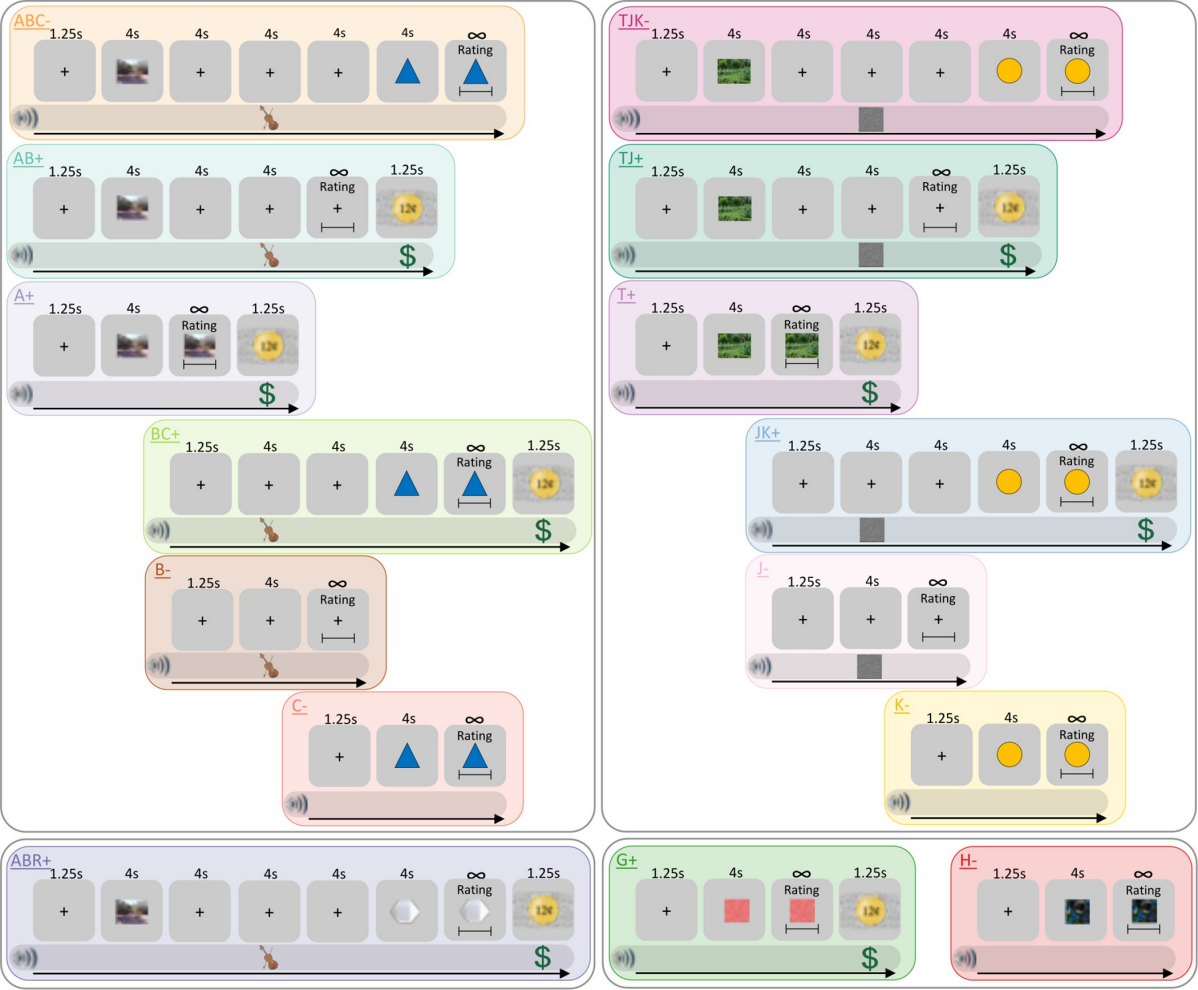
Experiment 1 (2^nd^-Order Negative Occasion Setting) Trial Design. Each colored box represents a trial type. Gray boxes represent what was shown visually on screen. Inter-trial intervals (ITIs) and inter-stimulus intervals (ISIs) included a gray screen with a fixation cross (“+”). Duration of each trial component is shown at top of each trial type. Rating slide is shown in abbreviated form, and visual analog scale was used to rate US Expectancy. Images of nature scenes, shapes, and auditory stimuli indicate experimental stimuli (i.e., CSs, occasion setters). Auditory stimuli are indicated below slides in horizontal auditory band. Violin symbol indicates violin sound, static screen indicates white noise, and dollar sign indicates cash register sound. None of the auditory symbols were shown on screen during the experiment. Gold coin indicates monetary reward (US). Black arrow pointing to the right for each trial type indicates chronological component sequence during trials. All stimuli are counterbalanced across participants within stimulus category: G/H (Unambiguous CSs), C/K (Ambiguous CSs), B/J (1^st^-order occasion setters), and A/T (2^nd^-order occasion setters). All trial types for Experiment 1 are shown except TJR+, which is identical to ABR+, except T and J stimuli are substituted for A and B. Experiment 2 (2^nd^-order POS) design is mirror image of Experiment 1 in which all trial types reinforced in Experiment 1 were not reinforced in Experiment 2, and all trial types not reinforced in Experiment 1 were reinforced in Experiment 2. The only exceptions are G+ and H-, which remained a CS+ and CS-, respectively, in each study. Images of violin, gold coin, confetti, fractals, and dollar sign were obtained from https://openclipart.org.

In both experiments, we trained three families of stimuli to produce direct associative learning, 1^st^-order occasion setting, and/or 2^nd^-order occasion setting (Fig 2). In Experiment 1, the stimulus families were the “ABC” stimuli, “TJK” stimuli, and “Direct Learning” stimuli (i.e., G+, H-, and R+). When multiple stimuli were presented within a trial, they were presented serially with inter-stimulus intervals to facilitate occasion setting [23]. Specifically, within the “ABC” stimulus family, C was trained as an ambiguous CS that predicted no US on its own but predicted the US when preceded by putative 1^st^-order positive occasion setter B (i.e., C-, BC+). Stimulus A acted as a putative 2^nd^-order negative occasion setter, so when A preceded BC, no US was delivered (i.e., ABC-). Additionally, B was non-reinforced on its own, and A acted as B’s putative 1^st^-order positive occasion setter (i.e., B-, AB+). Lastly, A was reinforced when presented on its own (i.e., A+). Thus, the stimulus contingencies were C-, BC+, ABC-, B-, AB+, A+. We hypothesized that stimulus A would have three hierarchical values (2^nd^-order negative occasion setter, 1^st^-order positive occasion setter, unambiguous CS+), B would have two hierarchical values (1^st^-order positive occasion setter, ambiguous CS-), and C would have one hierarchical value (i.e., ambiguous CS-). This training occurred prior to Transfer Test 1 and Transfer Test 2.

Also in Experiment 1, the “TJK” family was trained with identical meanings as the “ABC” family, substituting T for A, J for B, and K for C. The difference was that JK was trained *without* T before Transfer Test 1 (i.e., JK was trained only in 1^st^-order positive occasion setting), but JK was trained with T before Transfer Test 2 (i.e., in 2^nd^-order negative occasion setting). Thus, whereas the ABC family was trained in 2^nd^-order occasion setting prior to both transfer tests, the TJK family was trained just in 1^st^-order positive occasion setting before Transfer Test 1 (i.e., JK+, K-, J-) and was trained in 2^nd^-order negative occasion setting prior to Transfer Test 2 (i.e., K-, JK+, TJK-, J-, TJ+, T+). When testing A’s ability to transfer its 2^nd^-order negative occasion setting ability to JK+ (i.e., AJK), we predicted A would have less of an effect on JK+ at Transfer Test 1 than Transfer Test 2 because at Transfer Test 1, JK+ had not undergone 2^nd^-order occasion setting training yet. Lastly, G+ and H- were trained as an unambiguous CS+ and CS-, respectively, prior to Transfer Test 1. R was trained as an unambiguous CS+ prior to Transfer Test 1 as part of ABR+ and TJR+ (where “AB” and “TJ” had no influence on R’s reinforcement). This was done so that participants would not simply assume that A or T followed by two other stimuli would result in no US.

Due to the large number of trial types trained and tested, we conducted several sub-phases in uniform order across participants (see Table A in S7 Text for details). Within each sub-phase, each trial type received usually 5–10 training trials, and trial order was pseudo-randomized. During the first training phase (Training 1), participants were first trained in 1^st^-order positive occasion setting with the “JK” stimuli (J-, K-, JK+) and then the “BC” stimuli (B-, C-, BC+); they were then trained in 2^nd^-order negative occasion setting with the “ABC” stimuli (C-, BC+, ABC-, B-, AB+, A+); afterwards, they were trained in direct learning with ABR+ and then G+ and H-, where each of these stimuli/combinations always predicted the US (ABR+, G+) or its absence (H-). After this, participants engaged in the Reminder 1 training phase in which all previously trained stimuli were trained within one sub-phase.

Participants then conducted Transfer Test 1 in which we tested responding to the trained stimuli and novel combinations of stimuli. These novel combinations were designed to test whether 1^st^-order occasion setting and 2^nd^-order occasion setting were indeed learned, since an occasion setter will only affect stimuli that were previously trained in occasion setting (e.g., an occasion setter would not affect an unambiguous CS+ or CS-). We hypothesized that occasion setters would not transfer to stimuli that were not trained in that form of occasion setting (i.e., B and J would not transfer to H-; A would not transfer to JK+, G+, or H-; AB would not transfer to G+). This would be shown by more similar responding between the novel transfer combinations to the trained target than to the trained occasion setting combination (e.g., ABG would be more similar to G+ than ABC-; AJK would be more similar to JK+ than ABC-).

In the next training phase (Training 2), participants were trained with a new 2^nd^-order negative occasion setter (T) within the “TJK” family of stimuli. Note that “JK” was already trained in 1^st^-order positive occasion setting but not 2^nd^-order negative occasion setting; it would now be trained with T in 2^nd^-order negative occasion setting (TJK-, JK+, K-, J-, TJ+, T+). Participants were then trained in direct learning with TJR+ and engaged in the Reminder 2 training phase in which multiple trial types from the”ABC” and “TJK” families were trained within the same sub-phase (TJK-, JK+, K-, T+, ABC-, BC+, C-, A+).

Finally, the experiment concluded with Transfer Test 2, in which three trained trial types were tested (ABC-, TJK-, JK+) and one untrained combination testing 2^nd^-order negative occasion setting (AJK). Of critical importance: AJK was tested during both Transfer Test 1 and Transfer Test 2. At the time of Transfer Test 1, JK had only been trained in 1^st^-order positive occasion setting (i.e., it had not been trained with 2^nd^-order negative occasion setter T yet). During Transfer Test 1, we expected A would have little effect on JK. Conversely, after Transfer Test 1, JK was trained with 2^nd^-order negative occasion setter T, so we hypothesized A would have a relatively greater effect on JK during Transfer Test 2. If this occurred, it would provide strong evidence that A was indeed a 2^nd^-order negative occasion setter, as it would only affect JK *after* JK had been trained with a different 2^nd^-order negative occasion setter.

Experiment 2 followed the same structure as Experiment 1, but the US reinforcement contingencies were largely reversed. The “ABC” family became the “DEF” family, and reinforcement was reversed (i.e., C-, BC+, ABC- vs F+, EF-, DEF+; B- and AB+ vs E+ and DE-; A+ vs D-); the “TJK” family became the “UMN” family, and reinforcement was reversed; and the “Direct Learning” family largely remained as such, where G+ and H- remained the same, but R became S with reinforcement reversed (i.e., ABR+, TJR+ vs DES-, UMS-). For concision, we will not write the same details for Experiment 2 as mentioned above for Experiment 1. We instead refer the reader to Table A in S7 Text for those details, which are congruent across both experiments.

Additionally, we created a novel computational model to i) explain 2^nd^-order occasion setting for the first time (as well as 1^st^-order occasion setting and direct learning); ii) to posit, operationalize, and test stimulus ambiguity as a mechanism of higher-order learning; and iii) to predict the specific transfer effects that are observed in these forms of learning. Our computational model is related to our theoretical model in Fig 1. The details of our computational model are found in the Materials and Methods section, but we provide a brief overview here. Our computational model has separate learning variables for excitation and inhibition at each hierarchical level (six variables total: V, $\bar{V}$, P, N, P2, N2). Additionally, because occasion setters only affect CSs that have been trained in occasion setting, we have four learning variables representing the CS’s ability to be modulated in excitatory or inhibitory directions by 1^st^- or 2^nd^-order occasion setters ($\hat{P}$, $\hat{N}$, $\hat{P}2$, $\hat{N}2$). A special aspect of our model is that 1^st^-order occasion setting can only be learned if the CS is ambiguous (i.e., sometimes predicts the US; sometimes does not). This is reflected in one learning variable (γ1). Similarly, 2^nd^-order occasion setting can only be learned if the 1^st^-order occasion setter is also ambiguous (sometimes excites the CS/US association; sometimes inhibits it). This is reflected in one learning variable (γ2). Our model also has two free parameters: α (learning rate) and ί (leaky memory). The α parameter estimates the learning rate, and the ί parameter(s) set the degree of retention and functionally can move the individual’s asymptote of learning. If empirically supported, the goal of our model would be to provide insight into learning mechanisms and stimulate research on highly complex Pavlovian learning (see Materials and Methods for details on our computational model).

## Results

### Training

**Fig 3:**
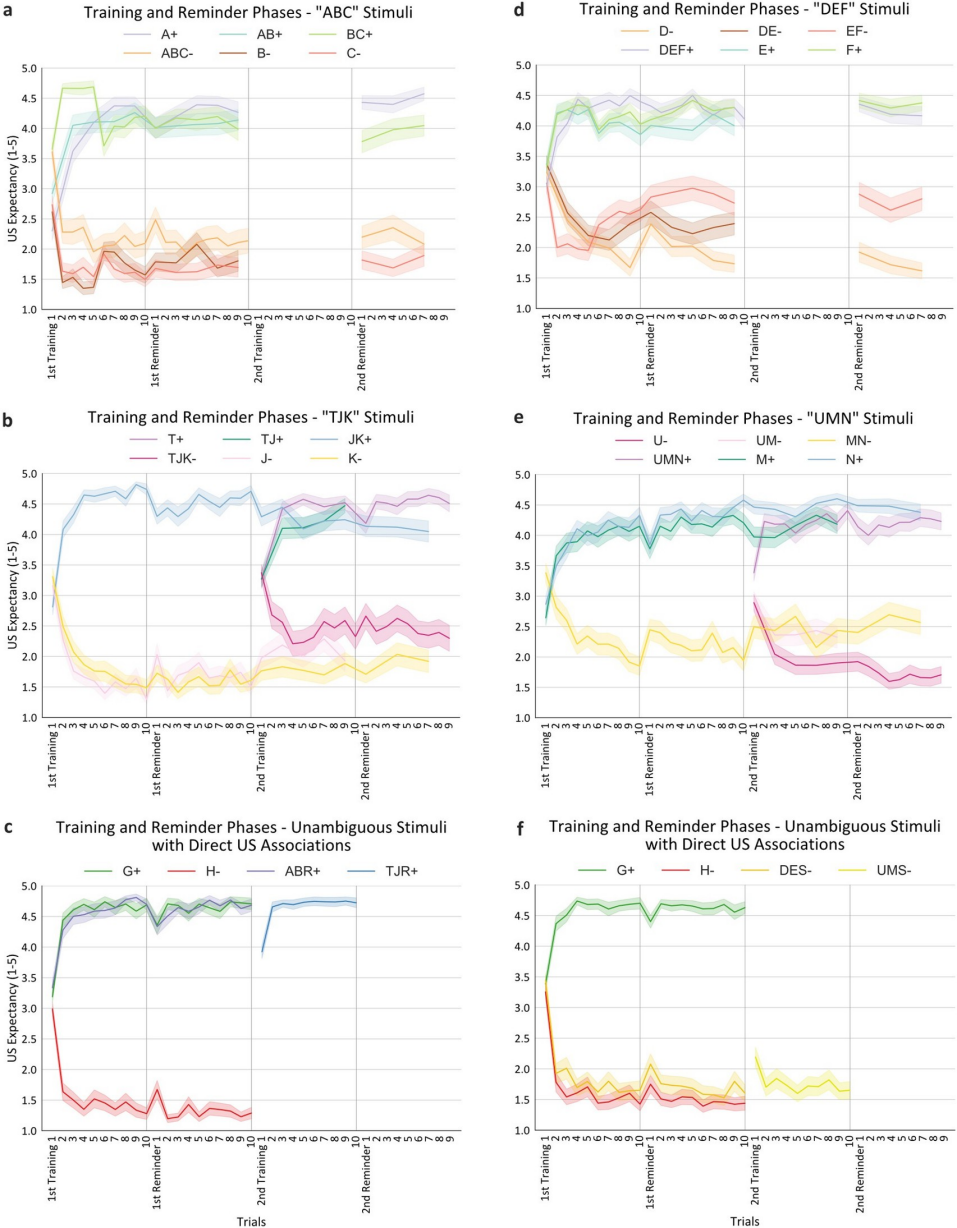
Experiment 1 and 2 Training Results. **a, b, c)** Experiment 1 Training results generally reflect direct CS/US associations, 1^st^-order positive occasion setting, and 2^nd^-order negative occasion setting. **d, e, f)** Experiment 2 Training results generally reflect direct CS/US associations, 1^st^-order negative occasion setting, and 2^nd^-order positive occasion setting. Congruent conditions/panels are displayed horizontally between experiments. Results in both experiments showed that participants correctly learned which stimuli were (non)reinforced. Error bands reflect standard error. Generally, “cool” colors (blues, greens, purples) indicate hypothesized higher values, whereas “warm” colors (reds, oranges, yellows) indicate hypothesized lower values. Additionally, because not all stimuli were trained in every phase of the experiment (e.g., the “ABC” and “DEF” stimuli were not trained during 2^nd^ Training), there are some empty spaces in the graphs of stimuli being shown either earlier or later in the experiment (e.g., G+ and H- were trained in the first half of the experiment). The reason that trial numbers vary between stimuli is to balance thoroughness of training and concision. During reminder phases, we wanted to remind participants of the most critical trial types relevant for the upcoming transfer test. For example, J- was not in the 2^nd^ Reminder phase because it was not essential to Transfer Test 2 (which focused on three-stimulus combinations, including ABC, AJK, and TJK). Additionally, the reader may notice that some stimuli (e.g., JK+) end prior to other stimuli (e.g., TJK-) during 2^nd^ Reminder. This is a visual artifact of the figure. The former stimuli received three trials of training during 2^nd^ Reminder (for concision purposes because they already received plenty of training beforehand), whereas the latter stimuli received nine trials because they had undergone less training. Thus, the figures show some stimuli (e.g., TJK-) having nine trials but others (e.g., JK+) having seven trials. Those stimuli end at seven trials in order to interpolate responding to them through the course of training and to scale their trial numbers for visual purposes. In short, this figure offers interpretable results of participants’ learning, and for detailed trial numbers, please see Table A in S7 Text.

Results from training phases are shown in Fig 3 (see Tables A and B in S4 Text for full statistical details). The critical test of reinforcement learning was the Reminder phases, as this was the end of each training section. This test was conducted by comparing the self-reported US expectancy ratings from each trial across stimuli. Overall, in both experiments, participants correctly learned which stimuli were reinforced and which were not for all trial types–direct associations, 1^st^-order occasion setting, and 2^nd^-order occasion setting. The most important and novel of these results was 2^nd^-order occasion setting: as hypothesized, 2^nd^-order occasion setting trial types had significantly lower (Experiment 1) and greater (Experiment 2) responding than their respective 1^st^-order occasion setting trial types during Reminder (e.g., Experiment 1: ABC- vs BC+; Experiment 2: UMN+ vs MN-; ps < .001).

### Transfer test

Transfer tests are used to determine whether occasion setting was indeed learned, in which a putative occasion setter is trained with one stimulus and is tested with a separately trained stimulus [1,2,7]. If the putative occasion setter only developed direct associative properties, it would summate with other CSs, producing responding equal to the sum of the presented stimuli. Conversely, true occasion setters transfer their occasion setting properties to stimuli that were separately trained in occasion setting, although this transfer is usually strong but incomplete. Thus, if a stimulus were a true occasion setter, we would expect it to have a greater effect on other stimuli trained in the same type of occasion setting than stimuli not trained in that form of occasion setting. We examine this in our transfer tests below.

See Tables A and B in S5 Text for details on statistical analyses. In total, all of our hypotheses were supported in Experiment 1, and most hypotheses were supported in Experiment 2. Each of the significant results below supporting our hypotheses survived Holm-Bonferroni correction for multiple comparisons [24].

**Fig 4:**
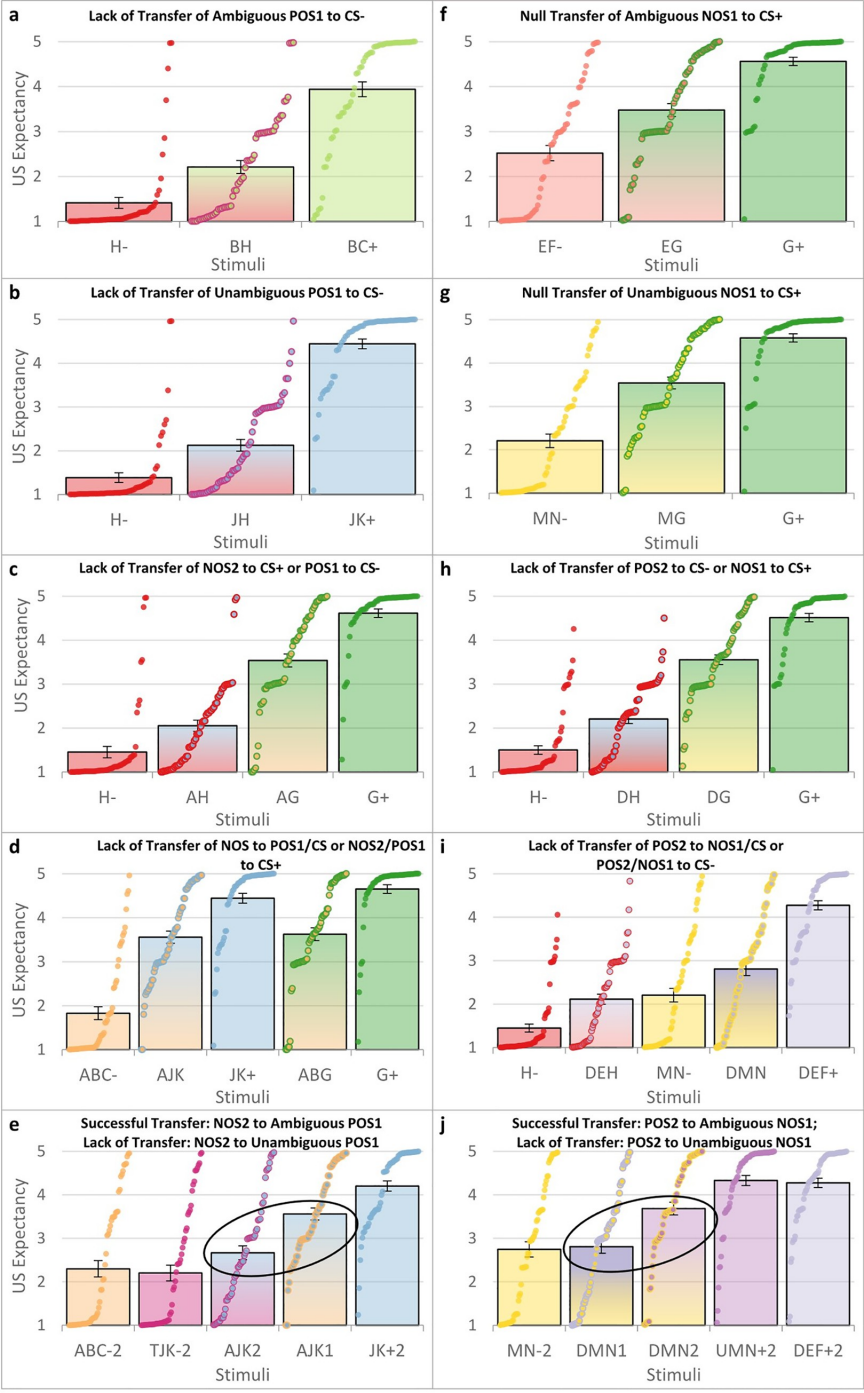
Experiment 1 and 2 Transfer Test Results. Panels **a-e** are from Experiment 1 (left column); panels **f-j** are from Experiment 2 (right column). Figure shows a bar plot (mean and standard error) with individual data points for each participant and each stimulus in ascending order. Bars with gradient colors and individual data points with two colors indicate novel transfer stimuli; solid bars and single-colored data points indicate trained stimuli. CS+ = excitatory conditional stimulus; CS- = inhibitory CS; POS1 = 1^st^-order positive occasion setter; NOS1 = 1^st^-order negative occasion setter; POS2 = 2^nd^-order POS; NOS2 = 2^nd^-order NOS. See main text for results details and references to panels. Main comparisons in panels **e** (AJK2 vs AJK1) and **j** (DMN2 vs DMN1) circled in **black ovals** and show that the 2^nd^-order occasion setters (A & D) transferred more strongly to the 1^st^-order occasion setter/CS combination after the combination was trained with a different 2^nd^-order occasion setter (AJK2, DMN2) than before (AJK1, DMN1).

Replicating previous research, we hypothesized that a 1^st^-order occasion setter would not affect an unambiguous CS [10,25,26]. To this end, we tested whether the novel stimulus combination was closer to one trained stimulus than another (e.g., whether the novel BH combination was closer to the trained H- or the trained BC+) because novel stimulus combinations would reasonably produce more uncertain responding than trained stimuli simply because they are novel (i.e., novel stimuli would have values closer to “3” on our 1–5 US expectancy scale, where 1 = “Certain No Bonus,” 3 = “Completely Uncertain,” and 5 = “Certain Yes Bonus”). Thus, we examined whether the novel stimulus combination was closer to one trained stimulus than another (see main text discussion and S5 Text for further discussion). This was tested three times in each experiment. In Experiment 1, all three 1^st^-order positive occasion setting transfer tests supported the hypotheses; in Experiment 2, one of three 1^st^-order negative occasion setting tests supported the hypotheses (constituting our only two null transfer test results across all hypotheses in both experiments). First, in Experiment 1, all three 1^st^-order occasion setting tests supported the hypotheses, as evidenced by BH (the transfer stimulus combination) having more similar responding to H- than BC+ (Fig 4A; p < .001), JH having more similar responding to H- than JK+ (Fig 4B; p < .001), and AH having more similar responding to H- than G+ (Fig 4C; p < .001). Experiment 2 showed that one of three 1^st^-order negative occasion setting transfer tests supported the hypotheses. Specifically, responding to EG was equidistant between EF and G+ (Fig 4F; p = .588), and responding to MG was equidistant between MN- and G+ (Fig 4G; p = .197). Conversely, our third test showed DG had more similar responding to G+ than H- (Fig 4H; p < .001), supporting the hypothesis.

Second, as part of our novel hypotheses, we hypothesized that 2^nd^-order occasion setters would only affect a CS if a 1^st^-order occasion setter were present; thus, we tested 2^nd^-order occasion setters on CSs in absence of 1^st^-order occasion setters. For example, during ABC trials, A was trained as a 2^nd^-order negative occasion setter with B as the 1^st^-order positive occasion setter and C as the CS. We would expect A to only affect a CS if a 1^st^-order occasion setter were present (e.g., if “B” were present from ABC). We thus tested the 2^nd^-order occasion setter in absence of a 1^st^-order occasion setter with a separately trained CS that had a learning value opposite to the 2^nd^-order occasion setter (i.e., 2^nd^-order negative occasion setter A (inhibitory) tested with G+ (excitatory); 2^nd^-order positive occasion setter D (excitatory) tested with a H- (inhibitory)). Results supported this hypothesis in both experiments. In Experiment 1, AG showed more similar responding to G+ than H- (Fig 4C; p < .001), and in Experiment 2, DH showed more similar responding to H- than G+ (Fig 4H; p < .001). This suggests that the 2^nd^-order occasion setters had minimal effects on the CSs in absence of 1^st^-order occasion setters.

Third, one of our critical novel tests was whether a 2^nd^-order occasion setter would affect *unambiguous* lower-order stimuli not trained in 2^nd^-order occasion setting; we hypothesized it would not. This was assessed using i) the trained 2^nd^-order occasion setter/1^st^-order occasion setter combination with an unambiguous CS (e.g., testing AB with G+ in ABG), as well as testing ii) the 2^nd^-order occasion setter with a trained unambiguous 1^st^-order occasion setter/CS combination (e.g., testing A with JK+ in AJK). In each case and in both experiments, all hypotheses were supported. Specifically, in Experiment 1, AJK had more similar responding to JK+ than ABC- (Fig 4D; p = .005), and ABG had more similar responding to G+ than ABC- (p = .005). Congruently, in Experiment 2, DMN had more similar responding to MN- than DEF+ (Fig 4I; p = .002), and DEH had more similar responding to H- than DEF+ (p < .001).

Fourth, our other critical novel test was to evaluate whether the ability of 2^nd^-order occasion setters to affect lower-order stimuli depended on whether the lower-order stimuli were ambiguous and trained in 2^nd^-order occasion setting. In the previous paragraph, we demonstrated that 2^nd^-order occasion setters had little effect on lower-order 1^st^-order occasion setter/CS combinations that were unambiguous and not trained with a 2^nd^-order occasion setter (e.g., A tested with JK+ in AJK; D tested with MN- in DMN). In each experiment, we later trained those same 1^st^-order occasion setter/CS combinations with a 2^nd^-order occasion setter (i.e., JK+ was later trained with T in TJK-; MN- was later trained with U in UMN+). We then tested whether a different 2^nd^-order occasion setter could affect the 1^st^-order occasion setter/CS combination more than it did before–now that the 1^st^-order occasion setter/CS combination had been trained with a 2^nd^-order occasion setter. Thus, the exact same stimulus combinations (i.e., Experiment 1: AJK; Experiment 2: DMN) were each tested twice–before and after the 1^st^-order occasion setter/CS combinations (JK+, MN-) were trained with a 2^nd^-order occasion setter (T, U). We hypothesized there would be a greater effect of 2^nd^-order occasion setters (i.e., A, D) on the 1^st^-order occasion setter/CS combinations (JK+, MN-) after the latter was trained with a different 2^nd^-order occasion setter (T, U). This hypothesis was supported in both experiments. In Experiment 1, the 2^nd^-order negative occasion setter (A) had a greater effect on JK+ after JK was trained with T (i.e., TJK-) (Fig 4E; see AJK1 vs AJK2 comparison; p < .001). Congruently, in Experiment 2, the 2^nd^-order positive occasion setter (D) had a greater effect on MN- after MN was trained with U (i.e., UMN+) (Fig 4J; see DMN1 vs DMN2 comparison; p < .001).

### Computational modeling

The behavioral results above demonstrate that 2^nd^-order occasion setting was learned, and these behavioral results can be bolstered by further evaluation of the underlying learning processes using computational modeling. We tested a computational model that allowed occasion setters to impact US expectancy only if lower-order stimuli were ambiguous and trained with occasion setters. That is, the influence of 1^st^-order occasion setters on CSs was dependent on CS ambiguity and CS training with a 1^st^-order occasion setter, and the influence of 2^nd^-order occasion setters on 1^st^-order occasion setters and CSs was dependent on 1^st^-order occasion setter ambiguity, CS ambiguity, and the CS’s training with a 1^st^-order and 2^nd^-order occasion setter. We compared models limited to each hierarchical level: our full 2^nd^-order occasion setting model (which also included 1^st^-order occasion setting and direct associations), our 1^st^-order occasion setting model (which also included direct associations), and our direct associations model (i.e., direct excitation and direct inhibition).

### Parameter recovery

For each model, we simulated random learning rate (α) and leaky memory (ί) parameters and evaluated the models’ ability to estimate those parameters accurately. In short, α sets the learning rate, ί sets the retention rate, and ί was estimated after α. All models in both experiments showed high correlations between simulated and recovered parameters (rs > .939; see Fig A in S6 Text), indicating that we were able to accurately estimate individual subjects’ parameter values.

### Model fit

**Fig 5:**
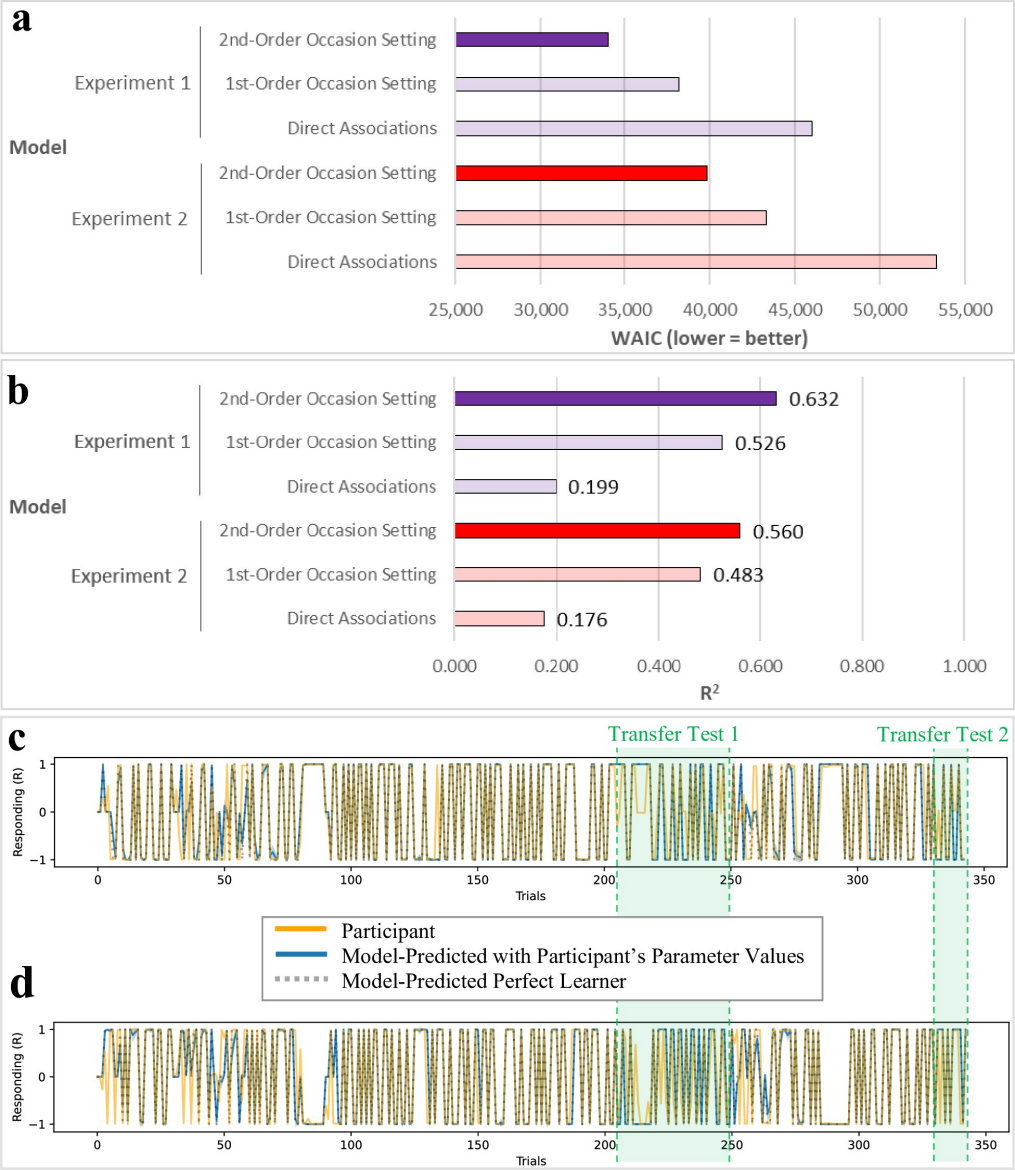
Computational Modeling Results. **a)** Model fit was determined with Watanabe-Akaike Information Criterion (WAIC), where lower scores indicate more accurate models. In both experiments, we tested three models of hierarchical learning: our direct associative learning model, our 1^st^-order occasion setting model (which also included direct associative learning), and our 2^nd^-order occasion setting model (which also included 1^st^-order occasion setting and direct associative learning). Results show that, in both experiments, the 2^nd^-order occasion setting model (bold colors) outperformed the 1^st^-order occasion setting model and direct associations model. **b)** As secondary/complementary results to our WAIC analyses, we also estimated median R^2^ for each model, finding that our 2^nd^-order occasion setting models had the greatest R^2^ (.632, .560). **c)** Experiment 1 exemplar participant responding, model-predicted responding, and “perfect learning” prediction for the 2^nd^-order occasion setting model. **d)** Experiment 2 exemplar participant responding, model-predicted responding, and “perfect learning” prediction for 2^nd^-order occasion setting model. Transfer Test 1 = trials 205–249 and Transfer Test 2 = trials 331–342, shown between the vertical green hashed lines; remaining trials were Training/Reminder.

We used Watanabe-Akaike Information Criterion (WAIC; [27]) to measure model fit. Our results showed that the 2^nd^-order occasion setting models outperformed the 1^st^-order occasion setting models, and both occasion setting models outperformed the direct associations models (Fig 5A). This suggests that our 2^nd^-order occasion setting model more closely resembles the underlying computations and learning process engaged in by the participants compared to the 1^st^-order occasion setting model or direct associative learning model. Secondary to the WAIC results, we estimated model R^2^ scores as a measure of our models’ explanatory power (Fig 5B). These results were fully congruent with the WAIC scores, where our 2^nd^-order occasion setting models had the highest R^2^ values at ≈ .6 (Experiment 1: R^2^ = .632; Experiment 2: R^2^ = .560). To illustrate our 2^nd^-order occasion setting model’s predictions, we plotted example participants’ real data and model-predicted data (Fig 5C and 5D). These examples show a high degree of overlap between our model’s predictions and the participants’ behavior.

## Discussion

This report investigates highly complex Pavlovian learning in order to experimentally model the complex associative learning that might occur in real-life circumstances. Pavlovian 1^st^-order occasion setting is a form of learning in which a 1^st^-order occasion setter signals whether a conditional stimulus (CS) will predict an outcome (the unconditional stimulus; US). Occasion setting occurs when the CS has a mixed (i.e., ambiguous) association in predicting the US and when the CS has been trained with a second stimulus that reduces the ambiguity (1^st^-order occasion setter). In our experiments, we examined the form of learning that occurs when both the CS and 1^st^-order occasion setter are ambiguous (i.e., both provide mixed signals of US (non)occurrence), positing that a third stimulus (the 2^nd^-order occasion setter) will signal how the 1^st^-order occasion setter modulates the CS/US association. We hypothesize three hierarchies of learning would be learned, in which the higher-order factors modulate the lower-order factors. In ascending order, these hierarchical levels are direct associative learning, 1^st^-order occasion setting, and 2^nd^-order occasion setting. Additionally, we created the first computational model predicting all three hierarchical levels of learning and evaluated its performance in two experiments.

A primary test of whether occasion setting was indeed learned is a transfer test, in which a putative occasion setter is tested with a separately trained stimulus. We would expect an occasion setter to only affect ambiguous lower-order stimuli trained in the same fashion with a different occasion setter. We conducted transfer tests in our experiments in which occasion setters were tested with i) the stimuli they were trained with and ii) separately trained stimuli. Our behavioral results can be summarized as the following: i) in most cases, a 1^st^-order occasion setter minimally affected responding to an unambiguous CS; ii) in all cases, a 2^nd^-order occasion setter minimally affected responding to an unambiguous CS or an unambiguous 1^st^-order occasion setter / CS combination; iii) in all cases, a 2^nd^-order occasion setter affected responding to an ambiguous 1^st^-order occasion setter / CS combination in the hypothesized direction; iv) the successful transfer of the 2^nd^-order occasion setter to the 1^st^-order occasion setter / CS combination was present but incomplete, which is the expected effect since occasion setters maximally affect the CS/US association they were trained with and have less effect on separately trained stimuli [1,7,14,15]; and v) participants learned that a given stimulus could have both excitatory and inhibitory meanings across all three hierarchies (direct learning, 1^st^-order occasion setting, and 2^nd^-order occasion setting). Our behavioral results were bolstered by the computational modeling results, which showed that the 2^nd^-order occasion setting model provided better fit (i.e., WAIC score) than the 1^st^-order occasion setting or direct associations model and, as a secondary/complementary analysis, had greater explanatory power (R^2^ ≈ .6). Integrating the behavioral and computational results, the evidence supports that 2^nd^-order occasion setting was learned and that our model is an accurate depiction of 2^nd^-order occasion setting.

In our theoretical and computational model, lower-order stimulus ambiguity (i.e., an inconsistent association between a stimulus and the US) is the gateway to higher-order learning; without ambiguity, occasion setting will not be learned (see Materials and Methods for computational model details). It is important to note that lower-order stimulus ambiguity is necessary but not sufficient for 1^st^-order or 2^nd^-order occasion setting to occur. In our model, the ambiguous CS must also be trained with a 1^st^-order occasion setter or 2^nd^-order occasion setter in order for each form of learning to occur. This assumes that a discernable occasion setter is present and is salient enough to acquire a learning value (which was the case in our experiments). This is exemplified by conducting occasion setter transfer tests. In our case, we evaluated transfer to an unambiguous, consistently (non)reinforced CS (i.e., CS+, CS-), in which we predicted and observed low transfer in most cases [25,26] (see next paragraph for discussion). Additionally, we compared 2^nd^-order occasion setter transfer to a 1^st^-order occasion setter/CS combination prior to and after the latter’s training with a different 2^nd^-order occasion setter. We predicted greater transfer after vs before 2^nd^-order occasion setting training [1,7,13–15,28], which we observed in both experiments. Conversely, a partially reinforced CS with no discernable occasion setter will be much less affected by occasion setters [13,29]. Our computational model predicts these transfer effects by allowing the CS to obtain the ability to be affected by 1^st^- or 2^nd^-order occasion setters (i.e., $\hat{P}$, $\hat{N}$, $\hat{P}2$, $\hat{N}2$), which requires the CS to be ambiguous (γ1 and γ2). Thus, only stimuli that are ambiguous and trained with an occasion setter can be affected by an occasion setter of the same type. Furthermore, while we are not the first to posit stimulus ambiguity as being a requirement of occasion setting [1,3–5,30] or to experimentally assess it [31], we are the first to extend it to 2^nd^-order occasion setting, investigate its robustness across learning hierarchies, and to create a theoretical and computational model operationalizing CS ambiguity and 1^st^-order occasion setter ambiguity. The data from the present experiments supports our computational model and its unique approach to operationalizing CS ambiguity (i.e., CS ambiguity = direct excitation * direct inhibition) and 1^st^-order occasion setter ambiguity (i.e., 1^st^-order occasion setter ambiguity = 1^st^-order positive occasion setting * 1^st^-order negative occasion setting) as a requirement of higher-order learning.

Furthermore, our statistical approach during transfer tests was to assess whether responding to the novel transfer stimulus combination was closer to one trained stimulus/combination or the other trained stimulus/combination. As described two paragraphs above, the results largely supported our hypotheses. However, we observed differences in responding comparing the novel combinations of stimuli to the unambiguous trained stimuli that were theoretically hypothesized to have similar responding to each other (see S5 Text for statistical details). For example, H- was a trained unambiguous CS-, BC+ was trained as a 1^st^-order positive occasion setting combination (from B-, C-, BC+), and novel combination BH showed responding that was significantly closer to H- than BC+ but was greater than H- (Fig 4A). This effect was generally observed across most novel vs trained transfer test stimuli. A likely reason that the novel stimulus combinations (e.g., BH) were significantly different from the trained unambiguous stimulus(i) (e.g., H-) is that the novelty of the transfer test stimulus combinations could produce US expectancy ratings closer to the midpoint of “Completely Uncertain” [32], which is what we observed. This interpretation seems fairly intuitive: uncertainty will be greater with novel stimuli compared to well-trained stimuli. However, we did not include a separate manipulation in our experimental design to test whether the novelty of the stimulus combinations indeed is what produced this change in responding, so we cannot conclude this with certainty. A second possibility is that the occasion setters developed a small degree of direct associative properties with the US when presented in combination with their trained CSs. For example, perhaps “B” from BC+ acquired largely 1^st^-order positive occasion setting abilities in the BC+ combination and a small degree of direct excitation with the US in this combination, as well. This direct excitation could then summate/transfer when tested with novel CSs (e.g., when H- was tested with B as BH). A third possibility is that the occasion setters had a small degree of transfer of their occasion setting properties to the unambiguous stimuli. This could happen if our “unambiguous” stimuli developed some degree of ambiguity, such as through generalization of learning from stimuli that were experimentally designed to be ambiguous stimuli trained with occasion setters (e.g., generalization from ambiguous stimuli C or K to unambiguous stimuli H- or G+). This interpretation would be consistent the broader occasion setting model since it would mean that the properties of ambiguous stimuli trained with occasion setters might generalize to “unambiguous” stimuli and allow the latter to be relatively minorly influenced by an occasion setter. A fourth explanation generally relies on imperfect knowledge and awareness from the participants (i.e., human error). Even if the environment and stimuli are designed to be deterministically ambiguous or unambiguous (as in our experiments), participants’ experiences might be different, for example, if they forget certain associations or miscredit which stimulus/combination led to the US or not. These inconsistencies could produce ambiguity in unambiguous stimuli, resulting in partial transfer of occasion setting properties (e.g., mistakenly thinking that H- led to the US or G+ led to no US could make them somewhat ambiguous and possibly allow occasion setters to minorly influence responding to them). In total, each of the explanations above is plausible, and they are not mutually exclusive. Perhaps one or more of them contributed to the partial transfer effects we observed, and future experiments could be designed to distinguish between these explanations.

Moreover, we believe that the CS is the primary target stimulus when presented with occasion setters, so we posit that the real-time moment of effect of an occasion setter occurs during the CS. For example, when a 1^st^-order positive occasion setter was presented (which also functioned as a CS- in our experiment), responding to this stimulus by itself was inhibitory, but when it was presented with the CS it was trained with in 1^st^-order positive occasion setting, responding was excitatory. Conversely, when the 1^st^-order positive occasion setter was followed by a separately trained unambiguous CS-, responding was inhibitory, thus suggesting that the CS is the target stimulus that activates the form of responding. Indeed, this is consistent with previous literature showing that the CS (rather than the occasion setter) controls response form [33]. It is also unlikely that participants were responding to blocks of cues, as we would expect very little transfer of the 2^nd^-order occasion setter to the separately trained 1^st^-order occasion setter / CS combination. In both experiments, we found strong transfer when expected.

Additionally, our model theoretically claims that mixed CS/US reinforcement will lead to broadening of attention to other situational factors, contexts, or stimuli to disambiguate whether the CS will predict the US on a given trial (i.e., γ1; [3,5,34,35]); we theorize the same for 1^st^-order occasion setters when they send mixed modulatory signals of CS/US reinforcement (i.e., γ2). Thus, our model posits when a search for 1^st^- or 2^nd^-order occasion setters will begin, but it is less clear when the search will end. In our experiments, the occasion setters deterministically signaled whether lower-order stimuli would predict the US, and there was only one occasion setter that was trained with a given lower-order stimulus or stimulus combination, so the search could theoretically end with perfect predictive accuracy once the occasion setter was identified and fully trained. It is less clear what would happen in non-deterministic occasion setting or in which there are multiple occasion setters present across trials. We presume the search for additional occasion setters would not occur while a trained, deterministic occasion setter is present (i.e., the occasion setter would block learning to other potential occasion setters) [36,37]. However, for example, if a 1^st^-order positive occasion setter’s presence perfectly signals that the CS will predict the US, but the CS still receives some mixed reinforcement when presented alone, we presume that the individual will continue searching for other occasion setters that will disambiguate the CS alone (perhaps there is an additional 1^st^-order positive or negative occasion setter the individual has not found yet). Thus, we posit that while mixed reinforcement is ongoing, the search for occasion setters is possible or likely. However, real-world constraints (e.g., time demands, energy) or other factors (e.g., relative importance of fully disambiguating this CS/US association vs focusing on other CS/US associations or engaging in other behaviors/tasks; whether the individual believes it is even possible to deterministically or near-deterministically predict the US) will likely affect whether an individual chooses to search for and learn about occasion setters that disambiguate US (non)occurrence. Ultimately, if an individual determines there are no occasion setters that can disambiguate the CS any further, they may infer that the unexplained mixed reinforcement of the CS is simply due to partial reinforcement. Furthermore, our free parameters of learning rate (α) and memory retention (ί) estimate and apply constraints that may affect how quickly an individual ends their search for occasion setters. For example, an individual who learns quicky and remembers strongly that an occasion setter affects the CS/US association may end their search more quickly than someone who learns slowly and remembers poorly. Our experiments were not designed to test when/how the search for occasion setters concludes, but future research could test this, and doing so would be very applicable to real-world scenarios in which there are presumably many occasion setters and CSs and in which learning rate and memory retention will likely affect the search for occasion setters.

The present results provide numerous clinical implications across many disorders that have strong Pavlovian components (e.g., anxiety, substance use). For example, anxious individuals seem to have deficits in discriminating safety from danger with direct associations [38–40] and increased fear of 1^st^-order occasion setting compounds [8]. The latter could be due to working memory deficits in anxious individuals [41–43] since 1^st^- and 2^nd^-order occasion setting are presumably more demanding of working memory than direct associative learning (i.e., needing to remember if an occasion setter was presented or not). Anxious individuals also have elevated intolerance of uncertainty [44,45]. We define “uncertainty” as relative difficulty predicting US (non)occurrence from the presented stimulus(i), whereas “ambiguity” is a different construct in which the individual learns that the stimulus(i) has/have a mixed association with the US. Due to the relative complexity of learning occasion setting vs direct associations, perhaps individuals experience greater uncertainty during occasion setting training or experience the uncertainty for more trials due to the complexity and ambiguity of occasion setting. This may lead anxious individuals to have greater fear of occasion setting combinations or depressed individuals to expect less reward from occasion setting compounds [8]. Second, our model claims that 1^st^-order occasion setters can be ambiguous (as has been shown elsewhere; e.g., [9,21]), and it argues that CS responding will be minimal if the CS has direct inhibition, is presented with a 1^st^-order negative occasion setter, and is absent of 1^st^-order positive occasion setters. This is relevant for anxiety disorders, as conventional exposure therapy focuses primarily on direct CS+ extinction with some effort to increase 1^st^-order negative occasion setters via context variability [46], but there is no explicit emphasis on 1^st^-order positive occasion setter extinction. Extinction of 1^st^-order positive occasion setting has been investigated elsewhere [16,20,36], demonstrating that extinction of the 1^st^-order positive occasion setter with the CS together (as opposed to extinction with the CS alone or the 1^st^-order positive occasion setter alone) is required in order to reduce responding to the 1^st^-order positive occasion setter/CS combination. For example, conducting exposures to public speeches (CS) could reduce fear to the CS in absence of any positive occasion setters, but if the client gives a speech (CS) after a charismatic speaker (unextinguished 1^st^-order positive occasion setter), fear will increase. From our model’s perspective, when a 1^st^-order positive occasion setter is extinguished, a stimulus/context could become a 2^nd^-order negative occasion setter (if it is salient enough and valid). This is relevant for exposure therapy for anxiety disorders, as it suggests that exposure therapists should not only encourage CS extinction, but 1^st^-order positive occasion setter extinction, as well. Perhaps one approach using this example would be to conduct speeches (CS) after a charismatic speaker (1^st^-order positive occasion setter) in a variety of contexts/situations to extinguish both, though this awaits testing in clinical studies.

Furthermore, our computational model uses an elemental approach both conceptually and mathematically [47]. Each sound, image, or perceptual occurrence is a separate stimulus that can contain multiple learning values (e.g., V, $\bar{V}$, P, N, P2, N2; see model in Materials and Methods) that get expressed differently depending on the presence or absence of other stimuli. Other models exist, such as latent cause models [48,49], in which individuals do not learn the associations between stimuli (e.g., CS, US, occasion setters); rather, they learn that *latent causes* predict the presence or absence of the stimuli, including the US. While we are unaware of a latent cause model that can predict 2^nd^-order occasion setting, and while evaluating its utility is beyond the scope of this report, it would be interesting to explore in future work. On the one hand, perhaps latent cause models of 2^nd^-order occasion setting would be parsimonious since they tend to cluster stimuli together as part of the latent causes, obviating the need for multiple associative weights. On the other hand, latent cause models might be complex because 2^nd^-order occasion setting is inherently complex; the latent cause model could require individuals to infer multiple latent causes. For example, in Experiment 1, stimulus A predicted the US as a CS+ and 1^st^-order positive occasion setter but predicted the US’s absence as a 2^nd^-order negative occasion setter. The latent cause model would need to account for the multiple predictive abilities of each stimulus, presumably requiring multiple latent causes to be inferred. It is also unclear how a latent cause model would predict the pattern of occasion setting transfer effects that are known to occur [1,7].

Additionally, our model is capable of predicting partial reinforcement of CSs or occasion setters using the leaky memory [50] ί parameters. These parameters allow learning values to leak and to provide an estimate of the participants’ inferred reinforcement rate. While Experiments 1 and 2 did not have partial reinforcement, we used a separate database of 75%/25% reinforcement in 1^st^-order positive occasion setting (as opposed to the traditional 100%/0% reinforcement rate) to test the utility of these leaky memory parameters (see S8 Text). Our results showed that including the ί parameters provided better model fit than excluding them. We additionally included the ί parameters in Experiments 1 and 2 for completeness, which are the models presented in the main text.

Furthermore, our computational model has some assumptions and limitations worth discussing. First, it engages in trial-by-trial learning rather than real-time learning [51,52]. However, the goal of our model is not to evaluate learning in real-time but rather to explain 2^nd^-order occasion setting for the first time (and the lower-order learning hierarchies), to posit and operationalize stimulus ambiguity as a mechanism of higher-order learning, and to predict the specific transfer effects that are observed in these forms of learning. Including real-time learning would require even more parameters and further complicate the model. Second, our model assumes that a discernable occasion setter is present in order for occasion setting to be learned. In the present experiments, these stimuli were clearly identifiable since they were tangible and salient (i.e., sounds, images). However, in situations of less salient (but valid) occasion setters, there would likely be individual differences in whether participants would detect and learn about the occasion setters. Third, our model assumes that less salient stimuli that provide information about CS (non)reinforcement are occasion setters. In our experiments, stimuli were designated as CSs or occasion setters based on previous work demonstrating which situations lead to occasion setting vs direct associative learning–all converging on the signal validity of the putative occasion setter and its relative salience to the CS [1,2,23,53]. Thus, we specified that temporal distance from the US (non)occurrence was positively associated with learning hierarchy [53].

Lastly, the neurocircuitry of 1^st^-order occasion setting remains largely unknown [1], and research on the neurocircuitry of 2^nd^-order occasion setting is non-existent. Because the learning process of 2^nd^-order occasion setting is theoretically similar to 1^st^-order occasion setting, it seems more likely and parsimonious that both forms of learning involve the same brain regions. However, this awaits empirical testing. Based on the limited research investigating the neurocircuitry of 1^st^-order occasion setting, candidate brain regions include the entorhinal cortex (EC), hippocampus, subiculum, orbitofrontal cortex (OFC), basolateral amygdala (BLA), and ventromedial prefrontal cortex (vmPFC). In short, the EC is involved in learning about temporally separated cues (like in occasion setting) and communicates this with the hippocampus [54–61]. Lesioning both the hippocampus and subiculum prevents occasion setting learning [25,62–66]. The OFC and BLA work together to flexibly learn changing associations between stimuli [1,67–82], and the OFC in particular has been shown to be active in occasion setting [83]. Furthermore, because the vmPFC is associated with extinction learning with simple CSs (which is arguably a form of 1^st^-order negative occasion setting) [84–88], it may also be associated with extinction of occasion setting (i.e., 2^nd^-order negative occasion setting). Neuroanatomically, the EC, hippocampus, subiculum, BLA, OFC, and vmPFC are connected [55,89–98], providing the structural ability to learn about changing cue meaning (BLA, OFC) for temporally separates cues (EC, hippocampus, subiculum) to produce occasion setting.

In conclusion, our experiments investigated highly complex associative learning and were the first to explicitly demonstrate the existence of 2^nd^-order occasion setting. We experimentally showed that lower-order stimulus ambiguity (i.e., conditional stimuli, 1^st^-order occasion setters) was necessary for higher-order learning (i.e., 1^st^- and 2^nd^-order occasion setting) to occur and that a 2^nd^-order occasion setter only transferred to a 1^st^-order occasion setter/CS combination when the latter was ambiguous and trained with a different 2^nd^-order occasion setter. Our stimuli were also trained in both excitatory and inhibitory directions across hierarchies, which would not be possible if occasion setting was not learned. These results in total are strong indications that 2^nd^-order occasion setting was indeed learned. Additionally, our computational model showed validity in predicting direct associations, 1^st^-order occasion setting, and 2^nd^-order occasion setting, and our 2^nd^-order occasion setting model outperformed simpler models based on 1^st^-order occasion setting and/or direct associative learning. These experiments are important to draw research attention to an additional layer of Pavlovian learning (2^nd^-order occasion setting), to more accurately model complex real-life associative learning, and to inform the treatment of disorders with strong Pavlovian components (e.g., anxiety, substance use).

## Materials and methods

### Ethics statement

This study was deemed exempt by the California Institute of Technology Institutional Review Board, and all participants provided written informed consent prior to commencing the study.

### Participants

Prolific [99] was used to recruit and collect human participant data online (Experiment 1: final N = 58; Experiment 2: final N = 67). Because 2^nd^-order occasion setting has not been investigated in a design like this, we did not have a strong basis for a power analysis. However, we pre-registered collecting 50–75 participants per experiment based on the strong effect size of 1^st^-order occasion setting as measured by US expectancy from transfer tests in our previous study (N = 80, d = .580 to 1.132; [8]). In the present experiments, we conducted post-hoc analyses of power for our most critical transfer tests (e.g., AJK2 vs AJK1, stimuli from t-tests comparing difference scores; see Tables A and B in S5 Text). In Experiment 1, the average power for our significant results was .9409 (range: .8257–1.0000); in Experiment 2, the average power for our significant results was .9768 (range: .8899–1.0000). Thus, our experiments were well-powered with our final sample sizes.

Participant eligibility criteria included being age 18–65, healthy or corrected vision, United States residents, English fluent, no hearing difficulties, and a Prolific approval rating of ≥95%; participants were only allowed to participate in one of the experiments. Across both experiments, demographics information included gender (53.60% female, 45.60% male, 0.80% agender), age (mean = 30.18, SD = 10.88, min = 18, max = 63), and ethnicity (10.40% Black or African-American, 8% Central/East Asian (e.g., Chinese, Japanese, Korean), 4.80% Hispanic or Latin(x), 6.40% South Asian (e.g., Indian, Pakistani, Sri Lankan), 62.40% White, and 8% Multiracial). Participants were paid $19.42 in Experiment 1 and $19.06 in Experiment 2 for completing the study. This amount was achieved by US presentations at the end of reinforced trials, where each US was a $0.12 USD increase in payment (as well as $2.50 for completing questionnaires).

Additionally, prior to data collection, we pre-registered each study (Experiment 1: https://osf.io/n2c6v, Experiment 2: https://osf.io/hxcfs). Based on our pre-registered exclusion criteria (i.e., automatic/invariant responding), we excluded one participant each from Experiment 1 and 2; also, an additional participant from Experiment 2 was excluded because of technical difficulties.

### Design

There were no between-subjects conditions; within-subjects conditions included trial number and stimuli with direct associations with the US (i.e., CSs), 1^st^-order occasion setters, and 2^nd^-order occasion setters. Experiment 1 was a 2^nd^-order negative occasion setting design, which included 1^st^-order positive occasion setting and 2^nd^-order negative occasion setting. Experiment 2 was a 2^nd^-order positive occasion setting design, which included 1^st^-order negative occasion setting and 2^nd^-order positive occasion setting. Thus, Experiments 1 and 2 are mirror opposites of each other. Both experiments included CSs with direct associations with the US. Our dependent variable was US expectancy, measured at the end of every trial.

### Materials and apparatus

The Pavlovian conditioning procedure was programmed using PsychoPy 2020.1.3. All learning stimuli (CSs, 1^st^-order occasion setters, 2^nd^-order occasion setters) were 4sec audio or visual stimuli. When multiple stimuli were presented within the same trial, they were presented serially with a 4sec inter-stimulus interval (ISI) between them. Serial presentation (rather than simultaneous presentation) is conducive to learning occasion setting rather than direct associations [23,100]. The US was a 1.25sec audio/visual stimulus showing an image of a gold coin with “12¢” written on it, confetti surrounding it, and an auditory cash register sound (i.e., “cha-ching!”). Inter-trial intervals (ITIs) were 1.25sec. ITIs and ISIs included a fixation cross, which was also displayed uninterrupted during audio stimuli. All trials ended with a US expectancy rating, which had no time constraint.

Unambiguous CSs (i.e., G+, H-; not trained with occasion setters) were images of fractals, ambiguous CSs (Experiment 1: C, K; Experiment 2: F, N; trained with occasion setters) were images of a blue triangle and orange circle, 1^st^-order occasion setters (Experiment 1: B, J; Experiment 2: E, M) were a violin sound and white noise sound, and 2^nd^-order occasion setters (Experiment 1: A, T; Experiment 2: D, U) were images of a desert and forest. Within each category, stimuli were counterbalanced across participants. Using different stimulus modalities (e.g., auditory, visual) between hierarchical levels facilitates distinction between direct and occasion setting learning [101]. Given that we only had two modalities to use, we made the 2^nd^-order occasion setting level visual (to distinguish from 1^st^-order occasion setting) but qualitatively different from the CS images (i.e., context images vs shapes/fractals). An additional unambiguous CS (Experiment 1: R; Experiment 2: S) was an image of a three-dimensional white gem; this stimulus was used to facilitate 2^nd^-order occasion setting (see S3 Text). Notably, the above list is the most hierarchically advanced function of each stimulus, but a given stimulus may have had more than one hierarchical meaning. For instance, each “1^st^-order occasion setter” listed above was also a CS with a direct association with the US, and this stimulus was modulated by a “2^nd^-order occasion setter” listed above, which acted as a 1^st^-order occasion setter in that case (e.g., Experiment 1: “B” was a 1^st^-order occasion setter (C-, BC+) but also a CS (B-, AB+); “A” was a 2^nd^-order negative occasion setter (ABC-, BC+, C-) but also a 1^st^-order positive occasion setter (B-, AB+) and a CS+ (A+)). This allowed us to test the specific hierarchical functions of each stimulus and determine whether independence between hierarchical levels was learned. Lastly, the following is a list of congruent stimuli between each Experiment (listed as Experiment 1/Experiment 2): A/D, B/E, C/F, J/M, K/N, R/S, T/U. G+ and H- were identical across both studies.

US expectancy. Participants used a visual analog scale to rate, “How certain are you that you are about to receive a bonus payment?” The values ranged from 1 = “Certain No Bonus”, 3 = “Completely Uncertain,” and 5 = “Certain Yes Bonus.” The visual analog scale did not display numerical values, but it displayed the anchors mentioned above. US expectancy was measured at the end of every trial using the mouse to click on the scale with unlimited time to respond.

### Procedure

Participants attended one experimental session online lasting approximately 1 hour 45 minutes, where they provided informed consent, completed questionnaires, and completed the Pavlovian learning experiment. In the experiments (Tables 1 and 2), participants were informed, “Your goal in this experiment is to learn which sounds and images predict receiving bonus payments.” During Training and Reminder phases, participants experienced US (non)reinforcement, which resulted in increases in their payment. Importantly, to maintain the Pavlovian nature of the experiment (rather than instrumental), participant responses did not affect their payment. During Transfer Test, participants were informed that they would not know whether they receive the US on these trials, which was accomplished by using an image of a curtain to cover the location on screen where the US image would otherwise occur and by muting the US sound. This curtain/muted modification was done so that no learning and no reinforcement/non-reinforcement would occur during Transfer Test, allowing us to test the underlying learning processes that occurred during training with many Transfer Test trial types (see S1 Text). We used the unambiguous CS+ and CS- as transfer test targets in many of our transfer tests based on previous experiments that exclusively used these stimuli as transfer test targets [8,25,26]. We additionally used the (un)ambiguous 1^st^-order occasion setter/CS combination as transfer test targets in our remaining transfer tests. Experimental phases followed a particular sequence, and within each sequence, stimulus presentation order was pseudo-randomized (see Table A in S7 Text for details on sequence and number of trials). In sum, Training/Reminder were conducted for participants to learn *which* stimuli predict (non)reinforcement, and Transfer Test was conducted to investigate *how* participants learned which stimuli predict (non)reinforcement.

**Table 1:** Summary of Experiment 1 Design. When three stimuli are listed (e.g., ABC-), 2nd-order occasion setting is hypothesized to be learned (except for ABR+ and TJR+). When two stimuli are listed (e.g., BC+), 1st-order occasion setting is hypothesized to be learned. When one stimulus is presented (e.g., C-), direct associative learning is hypothesized to be learned. All stimuli within a trial were presented serially. "+" indicates reinforcement; "-" indicates no reinforcement; "OS2" indicates 2nd-order occasion setting; "OS1" indicates 1st-order occasion setting; "Direct" indicates direct associative learning. Notably, the "ABC" family was trained in 2nd-order occasion setting before Transfer Test 1 and 2; the "TJK" family was only trained in 1st-order occasion setting before Transfer Test 1 but was trained in 2nd-order occasion setting before Transfer Test 2. This allowed the examination of whether "A" was indeed a 2nd-order occasion setter by testing "A" with "JK" before and after "JK" was trained with 2nd-order occasion setter "T." We predicted "A" would affect "JK" more strongly after "JK" was trained with "T" than before that training.

	Experiment Phase										
	Training 1/Reminder 1			Transfer Test 1			Training 2/Reminder 2			Transfer Test 2	
	OS2	OS1	Direct	OS2	OS1	Direct	OS2	OS1	Direct	OS2	OS1
**Stimulus Type**											
"ABC" Stimulus Family	ABC-	BC+	C-	ABC	BC		ABC-	BC+	C-	ABC	
		AB+	B-								
			A+						A+		
"TJK" Stimulus Family		JK+	K-		JK		TJK-	JK+	K-	TJK	JK
			J-					TJ+	J-		
									T+		
Unambiguous CSs			G+			G			TJR+		
			H-			H					
			ABR+			ABR					
Transfer Test Stimuli				ABG	BH						
				AJK	JH					AJK	
					AG						
					AH						

**Table 2:** Summary of Experiment 2 Design. When three stimuli are listed (e.g., DEF+), 2nd-order occasion setting is hypothesized to be learned (except for DES- and UMS-). When two stimuli are listed (e.g., EF-), 1st-order occasion setting is hypothesized to be learned. When one stimulus is presented (e.g., F+), direct associative learning is hypothesized to be learned. All stimuli within a trial were presented serially. "+" indicates reinforcement; "-" indicates no reinforcement; "OS2" indicates 2nd-order occasion setting; "OS1" indicates 1st-order occasion setting; "Direct" indicates direct associative learning. Notably, the "DEF" family was trained in 2nd-order occasion setting before Transfer Test 1 and 2; the "UMN" family was only trained in 1st-order occasion setting before Transfer Test 1 but was trained in 2nd-order occasion setting before Transfer Test 2. This allowed the examination of whether "D" was indeed a 2nd-order occasion setter by testing "D" with "MN" before and after "MN" was trained with 2nd-order occasion setter "U." We predicted "D" would affect "MN" more strongly after "MN" was trained with "U" than before that training.

	Experiment Phase										
	Training 1/Reminder 1			Transfer Test 1			Training 2/Reminder 2			Transfer Test 2	
	OS2	OS1	Direct	OS2	OS1	Direct	OS2	OS1	Direct	OS2	OS1
**Stimulus Type**											
"DEF" Stimulus Family	DEF+	EF-	F+	DEF	EF		DEF+	EF-	F+	DEF+	
		DE-	E+								
			D-						D-		
"UMN" Stimulus Family		MN-	N+		MN		UMN+	MN-	N+	UMN	MN
			M+					UM-	M+		
									U-		
Unambiguous CSs			G+			G					
			H-			H					
			DES-			DES			UMS-		
Transfer Test Stimuli				DEH	EG						
				DMN	MG					DMN	
					DG						
					DH						

### Data analysis

We used Stata 15.1 multilevel modeling for inferential statistics. For US expectancy during the Training phase, Level 1 predictors were Stimulus, Linear Slope, and Quadratic Slope. If the Quadratic Slope was not significant, it was removed from the model and re-run as a linear model. If the Linear Slope was not significant, it was removed and collapsed across Stimulus. For Transfer Test, the Level 1 predictor was Stimulus using the average of all three trials from a given block. We conducted multilevel modeling to examine whether one stimulus/compound was significantly different from another stimulus/compound. For analyses of the 2^nd^-order occasion setting compounds that were evaluated before and after 2^nd^-order occasion setting (i.e., AJK1 vs AJK2; DMN1 vs DMN2), we conducted multilevel modeling to determine if responding to AJK2 was lower than AJK1 and whether responding to DMN2 was greater than DMN1 (as hypothesized). For the remaining Transfer Test analyses, we used two sets of difference scores between the relevant stimuli and conducted paired samples t-tests on those difference scores. For example, in Experiment 1, when assessing whether the transfer test stimulus combination (BH) was significantly closer to the unambiguous CS (H-) than the putative 1^st^-order positive occasion setting compound (BC+) as hypothesized, we took a difference score of the higher value minus the lower value (e.g., BH minus H-; BC+ minus BH) and analyzed whether the difference scores were significantly different from each other. In this case, we examined whether BH minus H- was significantly smaller than BC+ minus BH; if so, this would indicate that BH was significantly closer to H- than BC+ as hypothesized. Within all analyses, we conducted Holm-Bonferroni corrections [24] to correct for multiple comparisons.

Furthermore, computational modeling was conducted using Python 3.7.6 to evaluate our theoretical model’s fit with the data using all trials. All models were fit using a hierarchical Bayesian approach, assuming subject-level parameters were drawn from group-level distributions, with parameters estimated using variational inference implemented in PyMC3 with 25,000 iterations. In our models, our free parameters were α (learning rate) and multiple ί parameters (leaky memory; this allows learning values to “leak” between trials and allows for the prediction of partial reinforcement). All other variables in the model were automatically calculated via the formulas, the stimuli presented, and the participants’ responses.

To evaluate parameter recovery, we separately simulated random α values ranging 0–1 and recovered them using our model with our sample size. We did the same with ί values depending on which model it was (see S6 Text for details). For all models, the correlation of simulated vs recovered α (rs > .999) and ί (rs > .939) parameters were very high (see Fig A in S6 Text). Model comparison was performed using Watanabe-Akaike Information Criterion (WAIC) scores [27], which provide a goodness of fit measure for Bayesian models, penalizing for increasing numbers of free parameters in the model (lower scores indicate better model fit). We chose our best-fitting model in each experiment based on the WAIC scores. To provide secondary/complementary model results, we calculated median R^2^ for each model using r2_score from the sklearn package.

### Our 2^nd^-Order occasion setting model

Ambiguity of the CS is one of the major purported mechanisms through which occasion setting as a learning process is theorized to occur [5,31,34,102,103]. Importantly, we view stimulus ambiguity as a dimensional and learned phenomenon–meaning, an individual needs to learn that a stimulus is ambiguous, and stimuli can vary in the degree to which they are ambiguous. According to the ambiguity hypothesis, a CS that always predicts the US has no ambiguity and therefore needs no other stimuli (i.e., occasion setters) to resolve which outcome it will predict on a given trial. In support, the evidence suggests that occasion setters can affect (i.e., transfer to) CSs that have undergone occasion setting training but have little-to-no effect on unambiguous CSs (e.g., CS+, CS-) (i.e., occasion setters generally do not affect responding to unambiguous CSs) [8,9,11,13,22,104–109]. CSs with partial US reinforcement but not trained with an obvious occasion setter are also ambiguous, but transfer of occasion setting to these CSs tends to not occur [13,29], presumably because the CS was not trained with an identifiable occasion setter. This suggests that CS ambiguity is necessary but not sufficient for occasion setting to occur. Another ambiguous stimulus is an extinguished CS+, where after having been trained with a particular outcome, the outcome is no longer delivered. Transfer of occasion setters to extinguished CSs is small but mixed, ranging from none [10,13] to partial [12,13,17]. This transfer is presumably greater than transfer to a partially reinforce CS because the extinguished CS+ was trained with an identifiable negative occasion setter (e.g., the physical or temporal extinction context), though generalization decrements between occasion setters can mitigate this transfer (e.g., if the other occasion setters are cues, such as a light or sound, rather than more diffuse physical or temporal extinction contexts). Lastly, transfer is highest between similar occasion setters trained with similar CSs and USs [7,10,13–15,17,18,28], ranging from partial [10,28] to complete [13,18,28]. Thus, a major way to determine whether occasion setting was indeed learned is to conduct transfer tests, where we would expect little-to-no transfer to unambiguous CSs and strong but not necessarily complete transfer to CSs that were trained with similar occasion setters and similar USs. Indeed, occasion setters are thought to have strong but incomplete transfer to CSs trained with other occasion setters, suggesting that occasion setters operate on the specific CS/US association they were trained with [1,2,7]. In contrast, if the putative occasion setter does not actually acquire occasion setting properties, we would expect simple summation of its direct associative value with other stimuli.

One foundational process through which occasion setting is learned is likely attentional. According to attentional theories of ambiguity [3,5,34,35], once a CS predicts more than one outcome, attention broadens to other stimuli or the context to find what will determine which outcome the CS predicts on a given trial. Because ambiguity is likely a learned and dimensional phenomenon, the degree to which attention is broadened to search for a disambiguating stimulus (i.e., an occasion setter) is also dimensional (i.e., for stimuli that are only slightly ambiguous, little effort is likely used to search for disambiguating stimuli; for stimuli that are highly ambiguous, more effort is likely used to search for disambiguating stimuli). We operationalize CS ambiguity as having a mixed direct association with the US (i.e., direct excitation * direct inhibition), and we operationalize 1^st^-order occasion setter ambiguity as having mixed modulation of the CS/US association in excitatory and inhibitory directions (i.e., 1^st^-order positive occasion setting * 1^st^-order negative occasion setting). This means that if a CS is ambiguous, a 1^st^-order occasion setter may modulate whether it predicts the US, and if a 1^st^-order occasion setter is ambiguous, a 2^nd^-order occasion setter may determine how the 1^st^-order occasion setter modulates the CS/US association.

**Fig 6:**
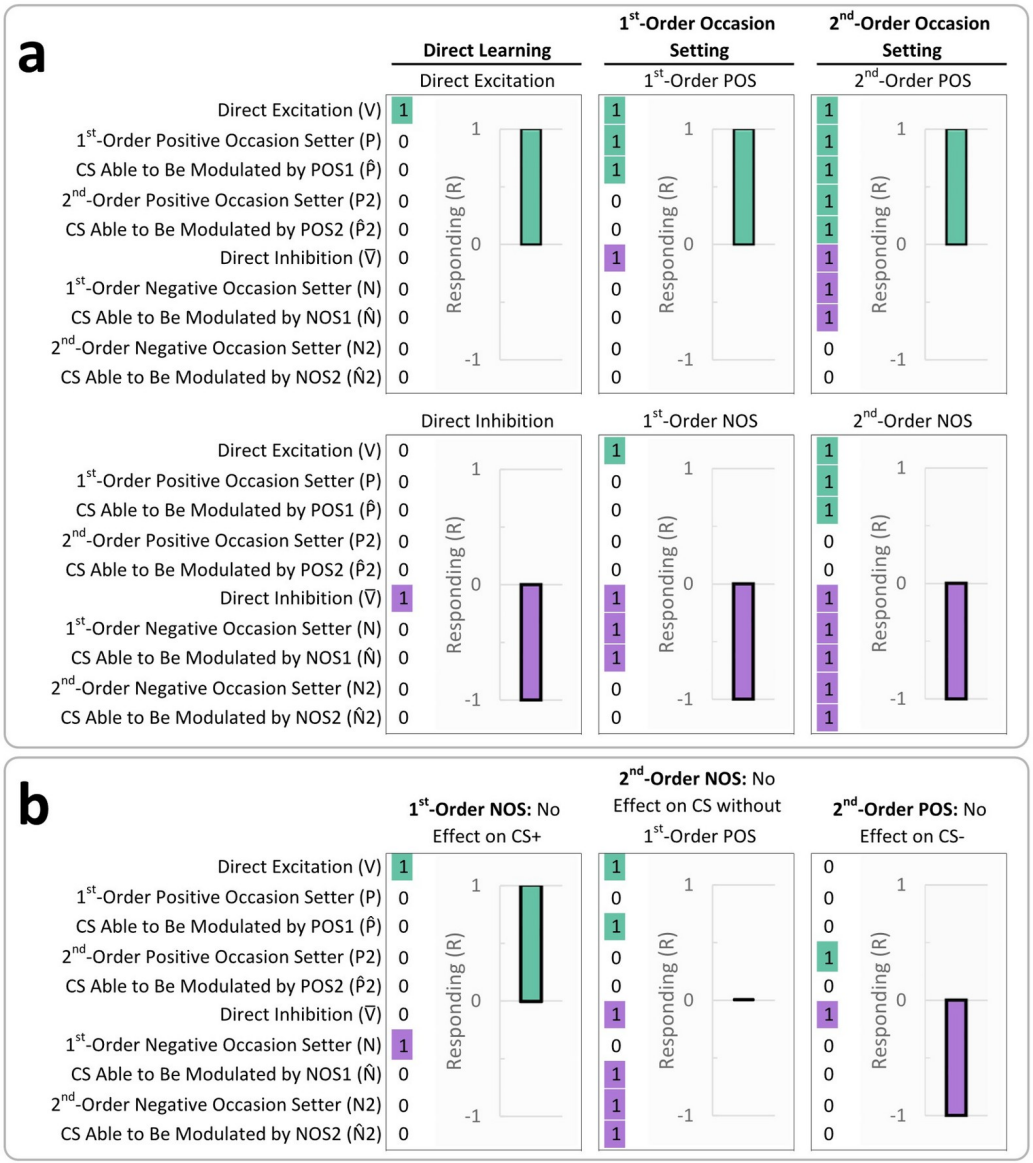
Examples of Formula Inputs and Outputs. The formula variables (e.g., V, $\bar{V}$, P) represent presence/absence of stimuli on a hypothetical trial and their training history. Bar graphs indicate predicted responding (i.e., R) to CS based on its training history and presence/absence of occasion setters. Left column provides names of formula variables. Across each row from these variables are values of 0 or 1, corresponding to the values of the variables’ names on the left. The values used by each figure are located in a column directly to the left of each figure. For example, the top-left figure shows a CS with 1 for direct excitation (V) and 0 for all other values (i.e., this is a CS+). POS = positive occasion setting; NOS = negative occasion setting. Excitation is color-coded as teal; inhibition is color-coded as purple. **a)** Examples of Direct Associative Learning and Successful Occasion Setting. We arranged inputs and outputs in a 2x3 grid, where the first column shows direct learning, the second column shows successful 1^st^-order occasion setting, and the third column shows successful 2^nd^-order occasion setting. First row shows excitatory responses, and second row shows inhibitory responses. **b)** Examples of Unsuccessful Occasion Setting. In bottom-left figure, we provide an example to demonstrate that 1^st^-order occasion setters do not affect responding if either direct excitation or direct inhibition are 0 (in our example, a 1^st^-order negative occasion setter does not affect a CS+, whose direct inhibition = 0). Congruently, 2^nd^-order occasion setters do not affect responding if any of the following are 0: direct excitation, direct inhibition, 1^st^-order positive occasion setting, or 1^st^-order negative occasion setting. As examples, in the bottom-middle plot, we show a 2^nd^-order negative occasion setter will not affect a CS unless an ambiguous 1^st^-order occasion setter is present (i.e., no 1^st^-order occasion setter is present, so P and N = 0). In our bottom-right plot, a 2^nd^-order positive occasion setter will not affect a CS- for multiple reasons, such as direct excitation = 0 and having no 1^st^-order occasion setter present (i.e., P and N = 0).

Our model follows a prediction error format [47] with several learning variables ranging 0 to 1 (see Table 3). The variables are ultimately combined into the “final” formula that predicts responding (i.e., R, ranging -1 to 1), in which a) direct excitation, 1^st^-order positive occasion setting, and 2^nd^-order positive occasion setting are added, and b) direct inhibition, 1^st^-order negative occasion setting, and 2^nd^-order negative occasion setting are subtracted. Many of these variables are all automatically calculated by the learning formulas and the inputted data. The only free parameters are α (learning rate) and ί (leaky memory). Additionally, see S1 Fig to download an interactive html file of our computational model, where the user can modify formula values to see the expected behavioral output. We suggest the reader uses this tool to more easily learn how our formulas work. For the reader’s convenience, we have provided example output of our computational model with varying levels of direct learning, 1^st^-order occasion setting, and 2^nd^-order occasion setting in the main text (see Fig 6). Note that 1^st^-order occasion setters will not affect responding unless both direct excitation and direct inhibition are > 0, and 2^nd^-order occasion setters will not affect responding unless direct excitation, direct inhibition, 1^st^-order positive occasion setting, and 1^st^-order negative occasion setting are all > 0.

**Table 3:** Formulas for Learning Direct Learning, 1^st^-Order Occasion Setting, and 2^nd^-Order Occasion Setting. In the table, subscripts are stimulus names (e.g., A, B, C; "sum" for all stimuli present on a trial); superscripts are trial numbers. For "R" formula, superscript "n" for all variables, and subscript "sum" for all variables except "R.” Formulas are arranged in column format for readability. Responding (R) formula ultimately predicts behavioral responding and learning. R operates by adding excitation and subtracting inhibition (formula in dark gray). Light gray columns highlight the similar variables used in the "recipe" across our learning formulas. Hierarchical control of a) 2^nd^-order occasion setting (2^nd^ OS) on 1^st^-order occasion setting (1^st^ OS) and b) 1^st^ OS on direct associative learning (i.e., CSs) is accomplished with modulation, in which the higher-order stimulus affects the lower-order stimuli’s signal of US (non)occurrence. The gateway to higher-order learning (from direct learning to 1^st^ OS, and from 1^st^ OS to 2^nd^ OS) is lower-order stimulus ambiguity. Mechanism through which 1^st^ OS is learned is γ1, which is the degree to which the CS is ambiguous (i.e., that the CS has both direct excitation and direct inhibition). If the present CS is unambiguous (e.g., only excitatory or only inhibitory), γ1 remains at 0, and no 1^st^ OS is learned. Once a given CS is ambiguous (i.e., the CS is both excitatory and inhibitory), γ1 becomes positive, allowing P and N to increase from zero and for 1^st^ OS to be learned. CS ambiguity is necessary but not sufficient for 1^st^-order occasion setting to be learned. In order for 1^st^ OS to be learned, the individual must also learn that the CS can be modulated by a 1^st^-order positive occasion setter ($\hat{P}$) or 1^st^-order negative occasion setter ($\hat{N}$). This will occur if a stimulus/context (i.e., the 1^st^-order occasion setter) provides information about the CS’s (non)reinforcement and if the stimulus/context is less salient than the CS ^e.g., 43^. $\hat{P}$ and $\hat{N}$ are values contained by the CS (rather than the 1^st^-order occasion setter), indicating the CS can be modulated by a 1^st^-order positive or negative occasion setter, respectively. Thus, a CS must be both ambiguous and trained with a 1^st^-order occasion setter in order to be modulated by other 1^st^-order occasion setters (e.g., a simple partially reinforced CS will not be affected by a 1^st^-order occasion setter because its $\hat{P}$ and $\hat{N}$ will equal 0, causing the 1^st^ OS terms in the R formula to equal 0).

	**Description**		**Formula**									**Range of Normal Values**
			R^n^ =									-1 to 1
	**Responding (R)**	**Direct Learning:**	(V - $\bar{V}$) +									
		**1^st^-order Occasion Setting:**	(P $\bar{V}$ $\hat{P}$)—(N V $\hat{N}$) +									
		**2^nd^-Order Occasion Setting:**	(P2 $\hat{P}2$ N V $\hat{N}$)—(N2 $\hat{N}2$ P $\bar{V}$ $\hat{P}$)									
**Direct Learning**	Direct Excitation (**V**)		ΔV_A_^n^	=		Λ^n^	α		(λ^n^ –	[V_sum_	^(n—1)^])	0 to 1
	Direct Inhibition ($\bar{V}$; "V Bar")		$\Delta {\bar{V}}_{A}{}^{n}$	=		${\bar{\Lambda }}^{n}$	α		(${\bar{\lambda }}^{n}$ –	[${\bar{V}}_{\mathrm{sum}}$	^(n—1)^])	0 to 1
	CS Ambiguity (**γ1**; "Gamma 1")		γ1_A_^n^	=	V_A_^n^ * $\Delta {\bar{V}}_{A}{}^{n}$							0 to 1
**1** ^**st**^ **-Order Occasion Setting**	1^st^-Order Positive Occasion Setting (**P**)		ΔP_B_^n^	=		Λ^n^	α	γ1_A_^n^	(λ^n^ –	[P_sum_	^(n—1)^])	0 to 1
	1^st^-Order Negative Occasion Setting (**N**)		ΔN_B_^n^	=		${\bar{\Lambda }}^{n}$	α	γ1_A_^n^	(${\bar{\lambda }}^{n}$ –	[N_sum_	^(n—1)^])	0 to 1
	Ability for CS to be Modulated by 1^st^-Order Positive Occasion Setter ($\hat{P}$)		$\Delta {\hat{P}}_{A}{}^{n}$	=		Λ^n^	α	γ1_A_^n^	(λ^n^ –	[${\hat{P}}_{\mathrm{sum}}$	^(n—1)^])	0 to 1
	Ability for CS to be Modulated by 1^st^-Order Negative Occasion Setter ($\hat{N}$)		$\Delta {\hat{N}}_{A}{}^{n}$	=		${\bar{\Lambda }}^{n}$	α	γ1_A_^n^	(${\bar{\lambda }}^{n}$ –	[${\hat{N}}_{\mathrm{sum}}$	^(n—1)^])	0 to 1
	1^st^-Order Occasion Setter Ambiguity (**γ2**; "Gamma 2")		γ2_B_^n^	=	P_B_^n^ * N_B_^n^							0 to 1
**2** ^**nd**^ **-Order Occasion Setting**	2^nd^-Order Positive Occasion Setting (**P2**)		ΔP2_C_^n^	=		Λ^n^	α	γ2_B_^n^	(λ^n^ –	[P2_sum_	^(n—1)^])	0 to 1
	2^nd^-Order Negative Occasion Setting (**N2**)		ΔN2_C_^n^	=		${\bar{\Lambda }}^{n}$	α	γ2_B_^n^	(${\bar{\lambda }}^{n}$ –	[N2_sum_	^(n—1)^])	0 to 1
	Ability for CS to be Modulated by 2^nd^-Order Positive Occasion Setter ($\hat{P}2$)		$\Delta \hat{P}2_{A}{}^{n}$	=		Λ^n^	α	γ2_B_^n^	(λ^n^ –	[$\hat{P}2_{\mathrm{sum}}$	^(n—1)^])	0 to 1
	Ability for CS to be Modulated by 2^nd^-Order Negative Occasion Setter ($\hat{N}2$)		$\Delta \hat{N}2_{A}{}^{n}$	=		${\bar{\Lambda }}^{n}$	α	γ2_B_^n^	(${\bar{\lambda }}^{n}$ –	[$\hat{N}2_{\mathrm{sum}}$	^(n—1)^])	0 to 1
**US-Related Variables**	US Presentation (**λ**; "Lambda")		λ^n^		Usually: if US occurs, λ^n^ = 1; if not, λ^n^ = 0							0 to 1
	Absence of Expected US ($\bar{\lambda }$; "Lambda Bar")		${\bar{\lambda }}^{n}$	=	V_sum_^(n—1)^							0 to 1
	Excitatory Learning Gating (**Λ**; "Big Lambda")		Λ^n^		If US occurs, Λ^n^ = 1; if not, Λ^n^ = 0							binary 0 or 1
	Inhibitory Learning Gating ($\bar{\Lambda }$; "Big Lambda Bar")		${\bar{\Lambda }}^{n}$		${\bar{\Lambda }}^{n}$ = 1 if Λ^n^ = 0 and V_sum_^n^ > 0; otherwise, ${\bar{\Lambda }}^{n}$ = 0							binary 0 or 1
**Free Parameters**	Learning Rate (**α**; "Alpha")		*Higher = Faster Learning Rate*									0 to 1
	Leaky Memory (**ί**; "iota")		*Higher = Greater Retention of Learning*									0 to 1

The above recipe of CS ambiguity and the CS’s ability to be modulated by a 1^st^-order occasion setter is extended to 2^nd^ OS. The mechanism through which 2^nd^ OS is learned is γ2, which is the degree to which a 1^st^-order occasion setter is ambiguous (i.e., that the 1^st^-order occasion setter is both a 1^st^-order positive and negative occasion setter). If the 1^st^-order occasion setter is unambiguous (e.g., either a positive or negative 1st-order occasion setter), γ2 remains at 0, and no 2nd OS is learned. Once a given 1st-order occasion setter is ambiguous (i.e., is both positive and negative), γ2 becomes positive, allowing P2 and N2 to increase from 0 and for 2^nd^ OS to be learned. 1^st^-order occasion setter ambiguity is necessary but not sufficient for 2^nd^ OS to be learned. In order for 2^nd^ OS to be learned, the individual must learn that the CS can be modulated by a 2^nd^-order positive occasion setter ($\hat{P}2$) or 2^nd^-order negative occasion setter ($\hat{N}2$). This will occur if a stimulus/context (i.e., the 2^nd^-order occasion setter) determines how the 1^st^-order occasion setter will modulate the CS’s (non)reinforcement. $\hat{P}2$ and $\hat{N}2$ are values contained by the CS, indicating the CS can be modulated by a 2^nd^-order positive or negative occasion setter, respectively.

The leaky memory variable (ί) is multiplied by each occasion setting Δ formula during each trial (e.g., P_B_^n^ = P_B_^(n-1)^ + ΔP_B_^n^ * ί_P, where ί_P is the ί for 1^st^-order positive occasion setting). This parameter allows for any reinforcement rate to be accurately estimated by our model. In this manuscript, we implemented ί values for excitation and inhibition at each occasion setting level and excluded ί for direct learning for parsimony. ί can be implemented at all hierarchical levels. An ί parameter value of 1 indicates that the individual retains 100% of that learning variable’s value; values lower than 1 reduce that learning variable’s value (e.g., if ί_P = .75, this will reduce P’s value to a maximum of .75), thereby enabling estimation of partial reinforcement. Employing multiple ί values across hierarchies, excitation, and inhibition allows for accurate prediction of participant responding.

The following is a more detailed description of the mechanics and assumptions of our model, with points 8 and 9 providing a verbal explanation of how responding (R) is conceptually and mathematically predicted.

1. Given that a stimulus adds unique predictive power with regards to whether the US will occur, we assume that stimulus salience is a determinant of whether the stimulus acquires occasion setting or direct associative properties [1,2,53]. Salience can be determined/manipulated in a number of ways [1,23,53], though the most common is to present occasion setters serially with their CSs, in which the CS’s onset is more proximal to the US, and the occasion setter’s onset is more distal [23]. In the present report, we assume that temporal distance from the US is positively associated with hierarchical learning (from direct learning to 1^st^-order occasion setting to 2^nd^-order occasion setting) if the more distant stimuli provide information regarding US occurrence beyond the information provided by the stimuli in the lower hierarchical levels. Thus, on a three-stimulus trial, the stimulus most distant to the US (non)occurrence is the 2^nd^-order occasion setter, the middle stimulus is the 1^st^-order occasion setter, and the most proximal stimulus is the CS. A similar pattern follows for two-stimulus trials (1^st^-order occasion setter is more distant from US than the CS). Although the present model assumes that temporal factors are critical in determining the learning accrued to each stimulus, this is not a real-time model. All that we assume here is that temporal ordering of stimulus’ presentation affects learning, but we do not manipulate the presentation time of each stimulus nor the inter-stimulus interval.
2. In our model, we use a delta rule of trial-by-trial learning with prediction error as a learning mechanism [47]. Prediction error is identical for our variable V as with the Rescorla-Wagner model’s V (i.e., λ–V_sum_) [47], where λ is the occurrence of the US (1 if US occurs, 0 if US does not occur; the US can also have values between 0 and 1 if using the same US with different intensities). Our model views excitation and inhibition as separate learning processes, and prediction error for inhibition is based on $\bar{\lambda }$ (i.e., the absence of an expected US). $\bar{\lambda }$ can only be greater than 0 if there is an expected US (i.e., if the stimuli present on the current trial have > 0 predictive value of the US). Our calculation is $\bar{\lambda }$ = V_sum_.
3. Our model includes gating variables which only allow excitation to be learned on reinforced trials (Λ; “big lambda”) and inhibition to be learned on non-reinforced trials ($\bar{\Lambda }$; “big lambda bar”). These variables are binary, where Λ = 1 if the US occurs, and $\bar{\Lambda }$ = 1 if no US occurs *and* if V_sum_ > 0 on that trial (i.e., there was at least some expectation the US would occur).
4. Our model has α (“alpha”) as a free learning rate parameter. It also has ί (“iota”) as a free leaky memory parameter [50]. The α parameter is multiplied *within* the Δ formulas to estimate the speed of prediction error learning (e.g., ΔP_B_^n^ = Λ^n^ * γ1_A_^n^ * α_B_ * (λ^n^–P_sum_^(n-1)^)). The ί parameters are multiplied *by* the Δ formulas to regulate how much excitation or inhibition is retained across trials at each hierarchical level (e.g., P_B_^n^ = P_B_^(n-1)^ + ΔP_B_^n^ * ί). Chronologically, α is calculated before ί, allowing α to set the learning rate and ί to maintain or reduce what was learned. Functionally, ί can maintain or lower the asymptote of predicted responding, allowing the asymptote to occur at any reinforcement rate (e.g., for 75% reinforcement, asymptote would be at .75 instead of a traditional asymptote of 1 for 100% reinforcement). Separate ίs can be used with each learning variable (e.g., V, $\bar{V}$, P, N, P2, N2) so they decrease independently, which enables different rates of partial reinforcement across excitation and inhibition at each hierarchical level. Thus, rather than each learning value reaching an asymptote of 1, ί allows them to asymptote at values between 0 and 1, which would reflect the individual’s perceived reinforcement rate of the stimulus(i) presented (see S8 Text for an example). In the main text experiments, we implemented ί parameters at the 1^st^- and 2^nd^-order occasion setting levels (i.e., P, N, P2, N2).
5. γ1 and γ2 are stimulus-specific ambiguity variables, where γ1 measures CS ambiguity and γ2 measures 1^st^-order occasion setter ambiguity. γ1 equals direct excitation multiplied by direct inhibition (i.e., V * $\bar{V}$), and γ2 equals 1^st^-order positive occasion setting multiplied by 1^st^-order negative occasion setting (i.e., P * N). We conceptualize ambiguity as a *learned* phenomenon (i.e., an individual must learn that a stimulus is ambiguous). In accordance with attentional models of ambiguity [5,31,34,102,103], we believe γ1 and γ2 result in increased attention to other stimuli, the background, context, or situational factors that will disambiguate which outcome the CS and/or 1^st^-order occasion setter will signal on a given trial. This allows attention to be directed towards concrete objects (e.g., other stimuli, contexts) or abstract concepts (e.g., time as a context, the absence of specific stimuli) to disambiguate the meaning of the CS or 1^st^-order occasion setter. γ1 is only included in the trial-by-trial Δ calculation for 1^st^-order occasion setting (i.e., ΔP, $\Delta \hat{P}$, ΔN, and $\Delta \hat{N}$), and γ2 is only included in the trial-by-trial Δ calculations to learn 2^nd^-order occasion setting (i.e., ΔP2, $\Delta \hat{P}2$, ΔN2, $\Delta \hat{N}2$). In other words, if a CS is ambiguous, 1^st^-order occasion setting can be learned. If 1^st^-order occasion setter is ambiguous, 2^nd^-order occasion setting can be learned. As long as γ1 = 0, no 1^st^-order occasion setting can be learned (and 2^nd^-order occasion setting cannot be learned); as long as γ2 = 0, no 2^nd^-order occasion setting can be learned. Importantly, whether stimulus B can become a 1^st^-order occasion setter depends on the γ1 for a *different* stimulus (e.g., stimulus A). For example, if stimulus A is ambiguous as a CS, then stimulus B can become its 1^st^-order occasion setter. The γ1 and γ2 formulas are calculated as the mean ambiguity of CSs or 1^st^-order occasion setters, respectively, present on a given trial, maintaining their 0–1 range. Similarly, whether stimulus C can become a 2^nd^-order occasion setter depends on the γ1 and γ2 for *different* stimulus (e.g., stimuli A and B); if A is ambiguous as a CS and B is ambiguous as a 1^st^-order occasion setter, then C can become a 2^nd^-order occasion setter. Additionally, while “ambiguity” is a learned phenomenon that a stimulus has a mixed prediction of the US, “uncertainty” is a different construct in which the individual has relative difficulty predicting the US from a stimulus(i). An ambiguous stimulus can be highly uncertain or have little-to-no uncertainty after training. For example, a 50% partially reinforced CS would be ambiguous (i.e., mixed association with the US) and relatively high in uncertainty (because we would not know which trial would lead to the US or not); conversely, an occasion-set CS (which is 100% reinforced on OS/CS trials and 0% reinforced on CS alone trials in our experiments) would be similarly ambiguous (i.e., mixed association with the US) but relatively low in uncertainty (because we can determine whether the CS will be reinforced based on the presence or absence of the occasion setter(s)).
6. Only CSs trained with a 1^st^-order occasion setter can be affected by a 1^st^-order occasion setter (i.e., $\hat{P}$ and $\hat{N}$), and only CSs trained with a 2^nd^-order occasion setter can be affected by a 2^nd^-order occasion setter (i.e., $\hat{P}2$ and $\hat{N}2$). This effect is most notably observed in transfer tests, where occasion setters will only affect a CS trained with a different occasion setter. Thus, our model predicts transfer only to CSs that are ambiguous and were trained with the specific type of occasion setter being tested.
7. Independence of direct associations and occasion setting has been repeatedly demonstrated across occasion setting studies [1,2,8]. Thus, in our model, direct excitation, direct inhibition, 1^st^order positive occasion setting, 1^st^-order negative occasion setting, 2^nd^-order positive occasion setting, and 2^nd^-order negative occasion setting are (largely) independent from each other–meaning, a given stimulus can simultaneously have any value of V, $\bar{V}$, P, N, P2, and N2. For example, stimulus A can be a direct excitor (V = 1), a 1^st^-order negative occasion setter (N = 1), and a 2^nd^-order positive occasion setter (P2 = 1). The only level of dependence between these variables in our model is that the rate at which inhibition is learned is dependent on the level of excitation (e.g., $\bar{\lambda }$ = V_sum_); otherwise, they are orthogonal.
8. The learning variables V, $\bar{V}$, P, N, P2, N2, $\hat{P}$, $\hat{N}$, $\hat{P}2$, and $\hat{N}2$ are computed together in a general “excitation minus inhibition” structure to produce a single output: R (i.e., responding). In the R formula, we first calculate direct excitation minus direct inhibition (i.e., V– $\bar{V}$). Functionally, this means direct excitation and direct inhibition will summate. To produce 1^st^-order positive occasion setting, we multiply 1^st^-order positive occasion setting (P) by direct inhibition ($\bar{V}$) and the CS’s ability to be modulated by a 1^st^-order positive occasion setter ($\hat{P}$) (i.e., $P*\bar{V}*\hat{P}$). Functionally, this means that 1^st^-order positive occasion setting will nullify direct inhibition if the CS has been trained with a 1^st^-order positive occasion setter, leading to an excitatory response. To produce 1^st^-order negative occasion setting, we multiply 1^st^-order negative occasion setting (N) by direct excitation (V) and the CS’s ability to be modulated by a 1^st^-order negative occasion setter ($\hat{N}$) (i.e., $N*V*\hat{N}$). Functionally, 1^st^-order negative occasion setting will nullify direct excitation if the CS has been trained with a 1^st^-order negative occasion setter, leading to an inhibitory response.
9. To produce 2^nd^-order positive occasion setting, we multiply 2^nd^-order positive occasion setting (P2) by the ability of the CS to be modulated by a 2^nd^-order positive occasion setter ($\hat{P}2$) and the entire 1^st^-order negative occasion setting term (i.e., $N*V*\hat{N}$), resulting in $P2*\hat{P}2*N*V*\hat{N}$. Functionally, this means that the 2^nd^-order positive occasion setter will nullify 1^st^-order negative occasion setting if the CS has been trained with a 2^nd^-order positive occasion setter, leading to an excitatory response. To produce 2^nd^-order negative occasion setting, we multiply 2^nd^-order negative occasion setting (N2) by the ability of the CS to be modulated by a 2^nd^-order negative occasion setter ($\hat{N}2$) and the entire 1^st^-order positive occasion setting term (i.e., $P*\bar{V}*\hat{P}$), resulting in $N2*\hat{N}2*P*\bar{V}*\hat{P}$. Functionally, this means that the 2^nd^-order negative occasion setter will nullify 1^st^-order positive occasion setting if the CS has been trained with a 2^nd^-order negative occasion setter, leading to an inhibitory response.
10. Within a typical 1^st^-order positive occasion setting paradigm (e.g., B➔A+, A-), 1^st^-order positive occasion setting as a learning process is purported to occur, but our model also predicts that 1^st^-order negative occasion setting will occur on trials with the CS alone. While B is the 1^st^-order positive occasion setter, the absence of B becomes a 1^st^-order negative occasion setter. The opposite occurs with 1^st^-order negative occasion setting (B➔A-, A+), where B is a 1^st^-order negative occasion setter, and the absence of B is a 1^st^-order positive occasion setter. The same occurs for 2^nd^-order occasion setting. The ability to learn that the absence of a stimulus is an occasion setter corresponds with attentional models of ambiguity [5,31,34,102,103], where attention can be directed to abstract contextual or situational factors to determine whether a stimulus will predict an outcome on a given trial. It also follows an intuitive verbal explanation for predicting the US (e.g., “when A is preceded by B, the US occurs, but when A is presented alone, the US does not occur”).
11. In the context of an experiment in which participants receive reinforcement depending on the trial type, we assume that inhibitory stimuli (i.e., CS-) acquire inhibition. This is based on studies in which the CS- acts as an control stimulus for the CS+ and develops inhibitory properties [8,110,111]. Thus, for CS-s that were never reinforced (or CSs that were initially not reinforced), we yoked their excitation to relevant CSs with excitation in order for the CS-s to acquire inhibition. Specifically, the yoked pairs in both experiments were H- to G+ (both experiments); the yoked pairs in Experiment 1 were B- to C and J- to K; and the yoked pairs in Experiment 2 were D- to F, U- to N, and S- to F. This produced the expected form of responding.

## Supporting information

S1 Fig Zbozinek et al 2^nd^-Order Occasion Setting Formulas. Interactive figure of our computational model, where the user can enter inputs for the learning variables and observe the model’s predicted outputs [112].
(HTML)

S1 Text Curtain during Transfer Test. (DOCX)

S2 Text Data Quality Assurance. (DOCX)

S3 Text Stimuli R and S. (DOCX)

S4 Text Detailed Training Results. Contains two tables: i) Table A. Experiment 1 Training and Reminder Phase Statistical Analyses, and ii) Table B. Experiment 2 Training and Reminder Phase Statistical Analyses.
(DOCX)

S5 Text Detailed Transfer Test Results. Contains two tables: i) Table A. Experiment 1 Transfer Test Statistical Analyses, and ii) Table B. Experiment 2 Transfer Test Statistical Analyses.
(DOCX)

S6 Text Simulated vs Recovered Parameters and ί Parameter Designation. Contains one figure: Fig A. Simulated and Recovered Learning Rate (α) and Leaky Memory (ί) Parameters.
(DOCX)

S7 Text Detailed Trial Sequence. Contains one table: Table A. Experiment Training Sequence.
(DOCX)

S8 Text Supplemental Experiment: 1^st^-Order Positive Occasion Setting with 75%/25% Reinforcement Rate. Contains two figures: i) Fig A. Supplementary Experiment Training Results, and ii) Fig B. Supplementary Experiment Real vs Model-Predicted Responding.
(DOCX)

S9 Text Additional Factors That Could Affect Whether Occasion Setting Is Learned. (DOCX)
