# Supplementary material for: Ambiguity drives higher-order Pavlovian learning
Source: PLoS Comput Biol. 2022 Sep 9;18(9):e1010410. doi: 10.1371/journal.pcbi.1010410 (PMC9491594; doi:10.1371/journal.pcbi.1010410)
Supplement: S1 Fig — Interactive figure of our computational model, where the user can enter inputs for the learning variables and observe the model’s predicted outputs [112]. (HTML) [file pcbi.1010410.s001.html]

Zbozinek et al 2nd-Order Occasion Setting Formulas 2
