# Supplementary material for: Ambiguity drives higher-order Pavlovian learning
Source: PLoS Comput Biol. 2022 Sep 9;18(9):e1010410. doi: 10.1371/journal.pcbi.1010410 (PMC9491594; doi:10.1371/journal.pcbi.1010410)
Supplement: S1 Text — (DOCX) [file pcbi.1010410.s002.docx]

**S1:** *Curtain during Transfer Test*

Transfer Test included many trials of trained and novel combinations of stimuli to assess the form of learning that occurred. Presenting these trials without reinforcement (as is typically done) would have likely produced extinction effects, reducing the validity of our results. Instead, we instructed participants that the stimulus/reward associations they learned during Training/Reminder would be maintained during Transfer Test, and they would receive reward appropriately. However, during Transfer Test, we covered up the audio/visual US with a curtain and muted the “cha-ching!” sound of monetary reward. Thus, participants did not know whether they received reward during Transfer Test trials. Participants were informed that, if they did receive reward, they would receive it at the end of the experiment. This procedure allowed us to conduct many tests during Transfer Test without any extinction effects.
