# Supplementary material for: Ambiguity drives higher-order Pavlovian learning
Source: PLoS Comput Biol. 2022 Sep 9;18(9):e1010410. doi: 10.1371/journal.pcbi.1010410 (PMC9491594; doi:10.1371/journal.pcbi.1010410)
Supplement: S2 Text — (DOCX) [file pcbi.1010410.s003.docx]

**S2:** *Data Quality Assurance*

Participants received three quizzes regarding important instructions in the experiment: prior to Training 1, prior to Transfer Test1 , and prior to Transfer Test 2. These quizzes were used to confirm that participants understood significant aspects of the study (e.g., the bonus payments are real). Participants needed a 100% on the quizzes in order to proceed in the experiment; scores lower than 100% prompted them to retake it until they got 100%. During Training and Reminder phases, participants experienced US reinforcement or omission. However, during Transfer Test phases, participants were instructed that everything they learned during training remains true about stimuli and bonus payments, but in the upcoming phase (i.e., Transfer Test), the US image will be covered with an image of a curtain, and the US cash register sound would be muted. Participants passed quizzes on these instructions prior to starting each Transfer Test.

Prior to any training, participants also heard a sound and adjusted their volume so the sound was being played at a comfortable volume. To ensure participants could hear experimental sounds throughout the experiment, at specific times (before Training 1, Reminder 1, and Training 2), participants received an audio quiz in which they had to correctly identify the audio sound being played (e.g., people playing table tennis, train passing by).
