# Supplementary material for: Ambiguity drives higher-order Pavlovian learning
Source: PLoS Comput Biol. 2022 Sep 9;18(9):e1010410. doi: 10.1371/journal.pcbi.1010410 (PMC9491594; doi:10.1371/journal.pcbi.1010410)
Supplement: S3 Text — (DOCX) [file pcbi.1010410.s004.docx]

**S3:** *Stimuli R and S*

Stimuli R and S were an unambiguous CS+ and CS-, respectively. However, R was always preceded by either AB or TJ, and S was always preceded by either DE or UM. In each of them, the preceding stimuli were irrelevant [1] – meaning, the preceding stimuli served no associative function as a CS or occasion setter. This is evidenced by participants’ responding to all presentations involving R and S nearly identically to separately trained and solo-presented unambiguous CS+ and CS-: G+ and H- (see Figure 3 in main text). As 2^nd^-order occasion setters, A and T always predicted no reward whenever followed by a 1^st^-order occasion setter and CS, and D and U always predicted reward whenever followed by a 1^st^-order occasion setter and CS. We included ABR+, TJR+, DES-, and UMS- in training so that participants would not assume that any trio presentation automatically meant reward (for D and U) or no reward (for A and T). This facilitated 2^nd^-order occasion setting learning.

1. Baker AG, Mackintosh NJ. Preexposure to the CS alone, US alone, or CS and US uncorrelated: Latent inhibition, blocking by context or learned irrelevance? Learn Motiv. 1979;10: 278–294. doi:10.1016/0023-9690(79)90034-1
