# Supplementary material for: Ambiguity drives higher-order Pavlovian learning
Source: PLoS Comput Biol. 2022 Sep 9;18(9):e1010410. doi: 10.1371/journal.pcbi.1010410 (PMC9491594; doi:10.1371/journal.pcbi.1010410)
Supplement: S4 Text — Contains two tables: i) Table A. Experiment 1 Training and Reminder Phase Statistical Analyses, and ii) Table B. Experiment 2 Training and Reminder Phase Statistical Analyses. (DOCX) [file pcbi.1010410.s005.docx]

**S4:** *Detailed Training Results*

For Experiment 1, see Table A for Training Phase results and Reminder Phase results. For Experiment 2, see Table B for Training Phase results and Reminder Phase results. To summarize: in both experiments, participants demonstrated successful training (i.e., learning which stimuli did and did not predict the US), as demonstrated by US expectancy ratings across Training and Reminder phases.

Specifically, in Experiment 1, ABC- had lower US expectancy than the relevant excitatory stimuli (A+, AB+, ABR+, and BC+) both by the end of Training Phase (ps < .002) and the end of Reminder Phase (ps < .001). Similarly, BC+ had greater US expectancy than relevant inhibitory stimuli (B-, C-) by the end of Training Phase (ps < .001) and the relevant inhibitory stimulus (ABC-) by the end of Reminder Phase (p < .001). Moreover, JK+ (prior to TJK- training) had greater US expectancy than relevant inhibitory stimuli (J-, K-) by the end of Training Phase (ps < .001) and greater US expectancy than TJK- by the end of Reminder Phase (after TJK- training) (ps < .001). G+ had significantly greater US expectancy than H- by the end of Training and Reminder Phases (ps < .001). Furthermore, TJK- had lower US expectancy than relevant excitatory stimuli (T+, TJ+, TJR+, JK+) by the end of Reminder Phase (ps < .001).

Similarly, in Experiment 2, DEF+ had greater US expectancy than the relevant inhibitory stimuli (D-, DE-, DES-, EF-) both by the end of Training Phase (ps < .001) and the end of Reminder Phase (ps < .001). Similarly, EF- had lower US expectancy than relevant excitatory stimuli (E+, F+) by the end of Training Phase (ps < .001) and the relevant excitatory stimulus (DEF+) by the end of Reminder Phase (p < .001). Moreover, MN- (prior to UMN+ training) had lower US expectancy than relevant excitatory stimuli (M+, N+) by the end of Training Phase (ps < .001) and lower US expectancy than UMN+ by the end of Reminder Phase (after UMN+ training) (ps < .001). G+ had significantly greater US expectancy than H- by the end of Training and Reminder Phases (ps < .001). Lastly, UMN+ had greater US expectancy than most relevant inhibitory stimuli (U-, UM-, MN-) by the end of Reminder Phase (ps < .011). Using the quadratic model, there was no significant difference between UMN+ and UMS- by the end of Training (p = .125), but using the linear model, UMN+ had significantly greater US expectancy than UMS- (mean difference: .175; Z = 2.71, p = .007).

**Table A. Experiment 1 Training and Reminder Phase Statistical Analyses.** 2nd NOS = 2nd-order negative occasion setting; 1st POS = 1st-order positive occasion setting. Within "Effects" (i.e., factors in statistical analysis), S = Stimulus, L = Linear Slope, Q = Quadratic Slope. When results of multiple models are presented (e.g., quadratic model, linear model, main effect of Stimulus model), they are indicated with highest-order factor in parentheses (e.g., "(Q)" for quadratic model). "Simple Effects" show most pertinent results. "Diff" indicates difference scores of stimuli in left column vs right column (e.g., value of ABC- minus values of A+, AB+, ABR+, etc.). Importantly, in order to indicate successful training, an effect involving Stimulus (main effect or interaction with slope) needed to be present in the Reminder Phases between relevant excitatory and inhibitory stimuli (e.g., stimuli within the "ABC" stimulus category, stimuli within the "TJK" stimulus category). Significant simple effects in **bold**.

**Table B. Experiment 2 Training and Reminder Phase Statistical Analyses.** 2nd POS = 2nd-order positive occasion setting; 1st NOS = 1st-order negative occasion setting. Within "Effects" (i.e., factors in statistical analysis), S = Stimulus, L = Linear Slope, Q = Quadratic Slope. When results of multiple models are presented (e.g., quadratic model, linear model, main effect of Stimulus model), they are indicated with highest-order factor in parentheses (e.g., "(Q)" for quadratic model)."Simple Effects" show most pertinent results. "Diff" indicates difference scores of stimuli in left column vs right column (e.g., value of DEF+ minus values of D-, DE-, DES-, etc.). Importantly, in order to indicate successful training, an effect involving Stimulus (main effect or interaction with slope) needed to be present in the Reminder Phases between relevant excitatory and inhibitory stimuli (e.g., stimuli within the "DEF" stimulus category, stimuli within the "UMN" stimulus category). Significant simple effects in **bold**.
