# Supplementary material for: Ambiguity drives higher-order Pavlovian learning
Source: PLoS Comput Biol. 2022 Sep 9;18(9):e1010410. doi: 10.1371/journal.pcbi.1010410 (PMC9491594; doi:10.1371/journal.pcbi.1010410)
Supplement: S5 Text — Contains two tables: i) Table A. Experiment 1 Transfer Test Statistical Analyses, and ii) Table B. Experiment 2 Transfer Test Statistical Analyses. (DOCX) [file pcbi.1010410.s006.docx]

**S5:** *Detailed Transfer Test Results*

See Table A for Experiment 1 and Table B for Experiment 2 Transfer Test results.

For positive 1^st^-order positive occasion setter to CS- transfer, BH, JH, and AH all had significantly greater responding than H- (Fig 4A-C; ps < .001). For 1^st^-order negative occasion setter to CS+ transfer, DG had significantly lower responding than G+ (Fig 4H; p < .001). For 2^nd^-order occasion setter to CS transfer, AG had significantly lower responding than G+ (Fig 4C; p < .001), and DH had significantly greater responding than H- (Fig 4H; p < .001). For 2^nd^-order negative occasion setter to lower-order stimuli transfer, AJK had significantly lower responding than JK+ (p < .001), and ABG had significantly lower responding than G+ (p < .001). For 2^nd^-order positive occasion setter to lower-order stimuli transfer, DMN had significantly greater responding than MN- (p < .001), and DEH had significantly greater responding than H- (p < .001).

Other studies also show mixed evidence regarding partial vs complete presence of transfer or lack of transfer [1–4]. Indeed, for each stimulus in our experiments, the transfer of the higher-order occasion setters to the lower-order stimuli showed modest increases in uncertainty compared to the trained. For example, in Experiment 1, responding to BH (transfer stimuli) was significantly greater than H- (trained stimulus) and significantly lower than BC+ (trained stimuli), but BH was significantly closer to H- than BC+. One very likely explanation is that the novelty of the compounds produced a degree of uncertainty irrespective of the underlying form of learning. This is completely reasonable, as any novel stimulus or stimulus combination could be expected to have greater uncertainty than a trained stimulus (assuming the trained stimulus was not something akin to a 50% partially reinforced stimulus). This would suggest that the differences in responding between a trained stimulus and novel transfer combination (e.g., H- vs BH) are due to the novelty of the latter – not due to any occasion-setting-based transfer effects. Another possibility is stimulus generalization: for example, stimulus H presumably shared some elements in common with stimulus C (e.g., both visual stimuli, size, location) and had functional overlap (e.g., both were CSs associated with the same US, same temporal proximity to US delivery/omission; [5]). The reason for the small degree of influence of B on H could be because the 1^st^-order positive occasion setter (B) was acting upon C’s and H’s common elements [2]. An additional possibility is that the putative occasion setters were in fact not occasion setters – or, they were occasion setters (e.g., 1^st^-order positive occasion setters) that also contained a small degree of direct associative properties in the same direction (e.g., direct excitation). However, this is less likely since these stimuli were trained to have direct associative properties in the opposite direction (e.g., “B” from B-, BC+) or 2^nd^-order occasion setting properties in opposite of the 1^st^-order occasion setting and direct associative properties (e.g., “A” from ABC-, AB+, A+). One more possibility is that the positive occasion setters acquired high incentive values [6] (i.e., that the positive occasion setters themselves were rewarding to experience) and that the negative occasion setters acquired low incentive values. While this may have affected our US expectancy data, it is unlikely this occurred since previous work has shown that incentive values persist even after the 1^st^-order positive occasion setter has been extinguished [6]. It is also unclear what the incentive values are for the stimuli that were trained as part of 2^nd^-order occasion setting; future work could examine this. Overall, the most likely explanation of our transfer test results is that the novelty of the transfer combinations produced a degree of uncertainty in responding to those stimuli because responding across various tests occurred in the direction we would expect for occasion setting.

Additionally, our 2^nd^-order occasion setting transfer results provide substantial support that 2^nd^-order occasion setting was indeed learned by providing a niche but expected powerful result if the stimulus were in fact a 2^nd^-order occasion setter. This specifically comes from two results. First, there was an increase in the effect of the 2^nd^-order occasion setter from before to after the 1^st^-order occasion setter/CS combination was trained with a different 2^nd^-order occasion setter (i.e., A’s influence on JK was greater from AJK1 to AJK2; D’s influence on MN was greater from DMN1 to DMN2, where “1” and “2” indicate before and after 2^nd^-order occasion setting training, respectively). Second, we would expect strong but incomplete transfer of 2^nd^-order occasion setting since occasion setters operate strongest on the CS/US association they were trained with and have decrements with other CSs or USs [7–11]. Thus, we would expect AJK (transfer combination) to have more uncertainty than TJK- or ABC- (trained combinations), and we would expect DMN (transfer combination) to have more uncertainty than DEF+ or UMN+ (trained combinations). We find this exact niche result, where AJK2’s responding is significantly more uncertain than ABC-2 and TJK-2, and DMN2’s responding is significantly more uncertain than DEF+2 and UMN+2 (Tables A and B). In sum, our results demonstrate a niche but powerful effect in which the 2^nd^-order occasion setters did not transfer to the 1^st^-order occasion setter/CS compound *before* the latter was trained with a 2^nd^-order occasion setter, but the former did transfer to the latter after the latter was trained with a 2^nd^-order occasion setter, and this transfer was incomplete (as expected). Thus, the results strongly suggest that 2^nd^-order occasion setting was indeed learned.

**Table A. Experiment 1 Transfer Test Statistical Analyses.** 2nd NOS = 2nd-order negative occasion setting; 1st POS = 1st-order positive occasion setting. "Simple Effects" show most pertinent results. "Diff" indicates difference scores of stimuli in left column vs right column (e.g., value of ABG minus G). Nested difference scores are the most relevant for testing transfer test hypotheses where present (e.g., (G - ABG) vs (ABG - ABC)). Main comparison for successful 2nd NOS at bottom is AJK2 vs AJK1. Significant simple effects surviving Holm-Bonferroni correction in **bold**.

**Table B. Experiment 2 Transfer Test Statistical Analyses.** 2nd POS = 2nd-order positive occasion setting; 1st NOS = 1st-order negative occasion setting. "Simple Effects" show most pertinent results. "Diff" indicates difference scores of stimuli in left column vs right column (e.g., value of DEH minus H). Nested difference scores are the most relevant for testing transfer test hypotheses where present (e.g., (DEH - H) vs (DEF - DEH)). Main comparison for successful 2nd POS at bottom is DMN 2 vs DMN1. Significant simple effects surviving Holm-Bonferroni correction in **bold**.

1. Holland PC. Transfer after serial feature positive discrimination training. Learn Motiv. 1986;17: 243–268. doi:10.1016/0023-9690(86)90013-5

2. Holland PC, Lamarre J. Transfer of inhibition after serial and simultaneous feature negative discrimination training. Learn Motiv. 1984;15: 219–243. doi:10.1016/0023-9690(84)90020-1

3. Morell JR, Davidson TL. Transfer across unconditioned stimuli in serial feature discrimination training. J Exp Psychol Anim Behav Process. 2002;28: 83–96. doi:10.1037/0097-7403.28.1.83

4. Davidson TL, Rescorla RA. Transfer of facilitation in the rat. Anim Learn Behav. 1986;14: 380–386. doi:10.3758/BF03200082

5. Honey RC, Hall G. Acquired equivalence and distinctiveness of cues. J Exp Psychol Anim Behav Process. 1989;15: 338–346. doi:10.1037/0097-7403.15.4.338

6. Fraser KM, Janak PH. Occasion setters attain incentive motivational value: implications for contextual influences on reward-seeking. Learn Mem. 2019;26: 291–298. doi:10.1101/lm.049320.119

7. Fraser KM, Holland PC. Occasion setting. Behav Neurosci. 2019;133: 145–175. doi:10.1037/bne0000306

8. Bonardi C. Occasion setting is specific to the CS–US association. Learn Motiv. 2007;38: 208–228. doi:10.1016/j.lmot.2006.08.003

9. Bonardi C, Ward-Robinson J. Occasion Setters: Specificity to the US and the CS–US Association. Learn Motiv. 2001;32: 349–366. doi:10.1006/lmot.2001.1089

10. Bonardi C, Robinson J, Jennings D. Can existing associative principles explain occasion setting? Some old ideas and some new data. Behav Processes. 2017;137: 5–18. doi:10.1016/j.beproc.2016.07.007

11. Trask S, Thrailkill EA, Bouton ME. Occasion setting, inhibition, and the contextual control of extinction in Pavlovian and instrumental (operant) learning. Behav Processes. 2017;137: 64–72. doi:10.1016/j.beproc.2016.10.003
