# Supplementary material for: Ambiguity drives higher-order Pavlovian learning
Source: PLoS Comput Biol. 2022 Sep 9;18(9):e1010410. doi: 10.1371/journal.pcbi.1010410 (PMC9491594; doi:10.1371/journal.pcbi.1010410)

**S6:** *Simulated vs Recovered Parameters and ί Parameter Designation*

Overall, every model showed excellent recovery of simulated α and ί parameters (Fig A), demonstrating that our models should have high accuracy in estimating α and ί parameters with real data. We designated which learning formula variables had ί parameters associated with them based on experimental design and model type. The goal of the ί parameters was to allow occasion setters to leak their learning values (P, N, P2, N2) in order to operate accurately under circumstances of partial reinforcement. Even though our main experiments had no partial reinforcement, we included ί parameters to provide the foundation for future experiments that may include partial reinforcement. For Experiment 1, our 2^nd^-order occasion setting model had ί parameters for 1^st^-order positive occasion setters (ί_P), 1^st^-order negative occasion setters (ί_N), the 1^st^-order negative occasion setting effect that occurs when a trained 1^st^-order positive occasion setters is absent (ί_Nabs), the 2^nd^-order positive occasion setting effect that occurs when a trained 2^nd^-order negative occasion setters is absent (ί_P2abs), and 2^nd^-order negative occasion setters (ί_N2). The 1^st^-order occasion setting model was a simplified version of this that excluded ί_P2abs and ί_N2. For Experient 2, our 2^nd^-order occasion setting model had ί_P, ί_N, the 1^st^-order positive occasion setting effect that occurs when a trained 1^st^-order negative occasion setters is absent (ί_Pabs), 2^nd^-order positive occasion setters (ί_P2), and the 2^nd^-order negative occasion setting effect that occurs when a trained 2^nd^-order positive occasion setters is absent (ί_N2abs). The 1^st^-order occasion setting model was a simplified version of this that excluded ί_P2 and ί_N2abs. The direct associations model had no ί parameters since that model had no occasion setting and since ί parameters were only implemented on the occasion setting hierarchies.

**a**

**b**

**Fig A. Simulated and Recovered Learning Rate (α) and Leaky Memory (ί) Parameters.** We randomized α and ί values from 0-1. We then conducted variational inference using 25,000 iterations to recover our randomly generated α and ί values with our experiment sample sizes. Correlations between simulated and recovered parameters in all models (including direct associations and 1^st^-order occasion setting models) were ≥ .939. Average correlation in 2^nd^-order occasion setting models was r = .961 (Experiment 1) and r = .975 (Experiment 2). **a)** Experiment 1 results. **b)** Experiment 2 results.


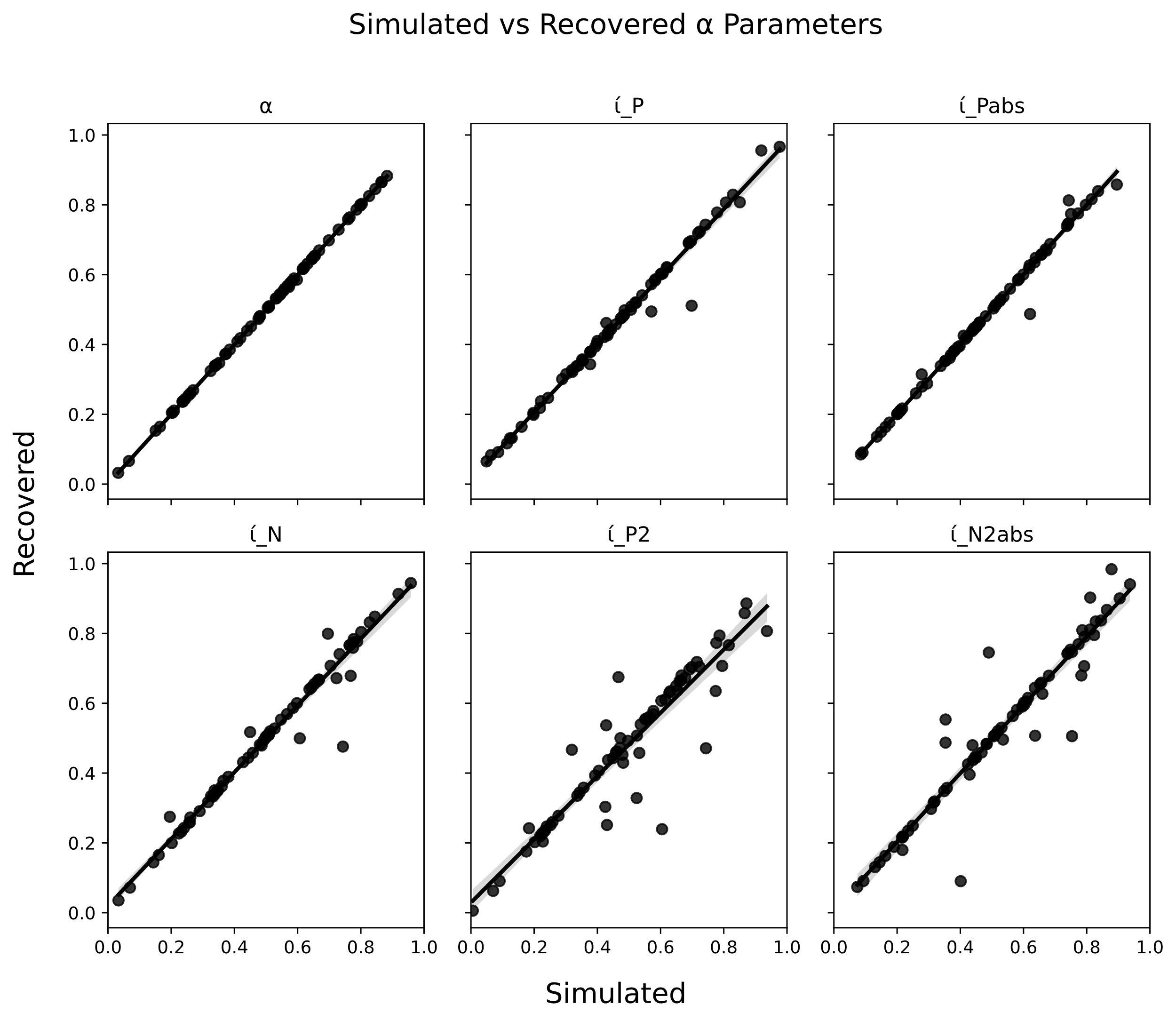

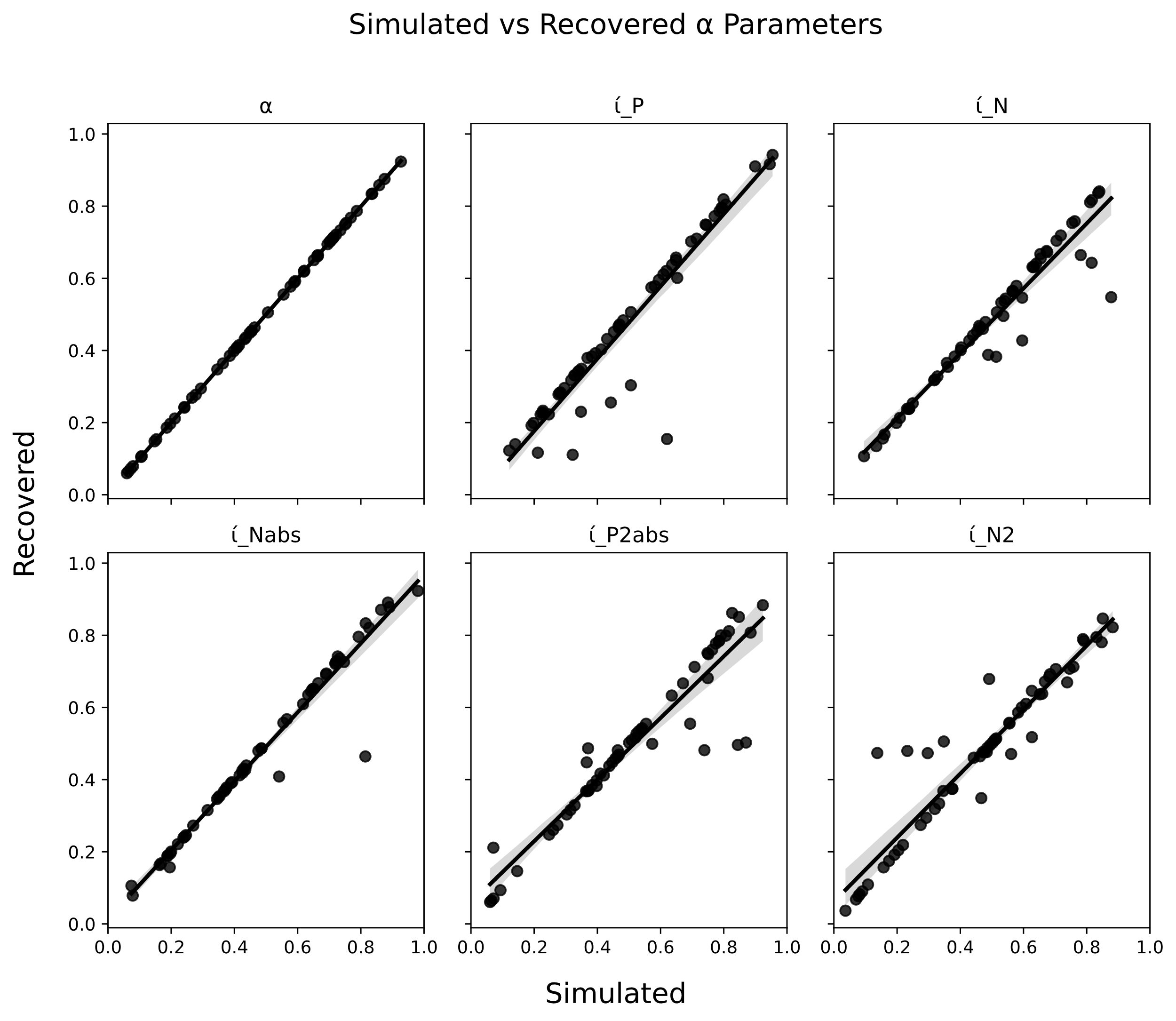

Supplement: S6 Text — Contains one figure: Fig A. Simulated and Recovered Learning Rate (α) and Leaky Memory (ί) Parameters. (DOCX) [file pcbi.1010410.s007.docx]
