# Supplementary material for: Ambiguity drives higher-order Pavlovian learning
Source: PLoS Comput Biol. 2022 Sep 9;18(9):e1010410. doi: 10.1371/journal.pcbi.1010410 (PMC9491594; doi:10.1371/journal.pcbi.1010410)
Supplement: S7 Text — Contains one table: Table A. Experiment Training Sequence. (DOCX) [file pcbi.1010410.s008.docx]

**S7:** *Detailed Trial Sequence*

**Table A. Experiment Training Sequence.** Experiments 1 and 2 are mirror images of each other in which reinforced and non-reinforced trials are swapped. Within each experiment, chronological training sequence occurs from top-left to bottom-right. Major phases include 1^st^ Training, 1^st^ Reminder, 1^st^ Transfer Test, 2^nd^ Training, 2^nd^ Reminder, and 2^nd^ Transfer Test. "NOS" and "POS" refer to negative and positive occasion setting, respectively. The association numbers (e.g., NOS1, NOS2) refer to 1^st^-order and 2^nd^-order occasion setting, respectively. Trial numbers for each stimulus are indicated; Transfer Tests include 3 trials of each stimulus per phase. Within each phase, trial sequence is randomized in minimal divisible blocks (e.g., 1^st^ Reminder: 2 trials each for ABC-, J-, etc. and 1 trial each of B-, C-, etc.). Within 1^st^ Transfer Test, we predict lack of transfer of occasion setters to lower-order unambiguous stimuli (i.e., 2^nd^ OS will not transfer to unambiguous OS1 or unambiguous CS). Within Transfer Test 2, we predict presence of transfer within 2^nd^-order occasion setting hierarchical level to ambiguous OS1, which is evaluated by comparing the same stimuli when OS1 was unambiguous (Transfer Test 1) and ambiguous (Transfer Test 2) (i.e., Experiment 1: AJK2 vs AJK1; Experiment 2: DMN2 vs DMN1).
