# Supplementary material for: Ambiguity drives higher-order Pavlovian learning
Source: PLoS Comput Biol. 2022 Sep 9;18(9):e1010410. doi: 10.1371/journal.pcbi.1010410 (PMC9491594; doi:10.1371/journal.pcbi.1010410)

**S8:** *Supplemental Experiment: 1^st^-Order Positive Occasion Setting with 75%/25% Reinforcement Rate*

The model in the main text is capable of predicting any form of partial reinforcement using the leaky memory parameter ί (i.e., “iota”). Here, we briefly present a supplementary experiment in which participants (N = 20) were trained in a 75%/25% reinforcement 1^st^-order positive occasion setting design. Specifically, the CS was 25%-reinforced when presented alone and 75%-reinforced when preceded by a putative 1^st^-order positive occasion setter (i.e., the OS). Two sets of stimuli were trained in this manner (OS1→CS1 (75% US), CS1 (25% US); OS2→CS2 (75% US), CS2 (25% US)). The US was the same audio-visual monetary US as in our 2^nd^-order occasion setting experiments but valued at $0.20 per US to provide a similar hourly payment rate. During Training, participants were trained in one stimulus arrangement first (e.g., OS1→CS1, CS1) and then the other arrangement (e.g., OS1→CS2, CS2) – the order of which was randomized. They then engaged in the Reminder phase, in which both stimulus groups were trained within the same phase. Trial order was pseudorandomized (no more than four of a given trial type could occur sequentially), occasion setters were a violin sound or trumpet sound (randomized), and CSs were a blue triangle or green star (randomized). Occasion setters, CSs, and the trace period between occasion setters and CSs were 5.1 sec. Our measure was US expectancy on a 1-5 scale like in the main text (1 = “Certain No Bonus”, 3 = “Completely Uncertain,” and 5 = “Certain Yes Bonus”) measured at the end of every trial.

Results showed that participants learned the reinforcement contingencies appropriately (Fig B). Using a multilevel model in which Stimulus (OS/CS, CS) and Trial (1-16) were Level 1 within-subjects factors, there was a main effect of Stimulus (χ^2^(1) = 185.50, p < .001), where the OS/CS combination had greater US expectancy across trials than the CS alone. There was no main effect of Trial (χ^2^(15) = 17.25, = .304) nor a Stimulus x Trial interaction (χ^2^(15) = 19.93, p = .175). For completeness, simple effects showed there was greater US expectancy during the OS/CS than CS alone on all trials (ps < .013) except trial 1 (p = .917).


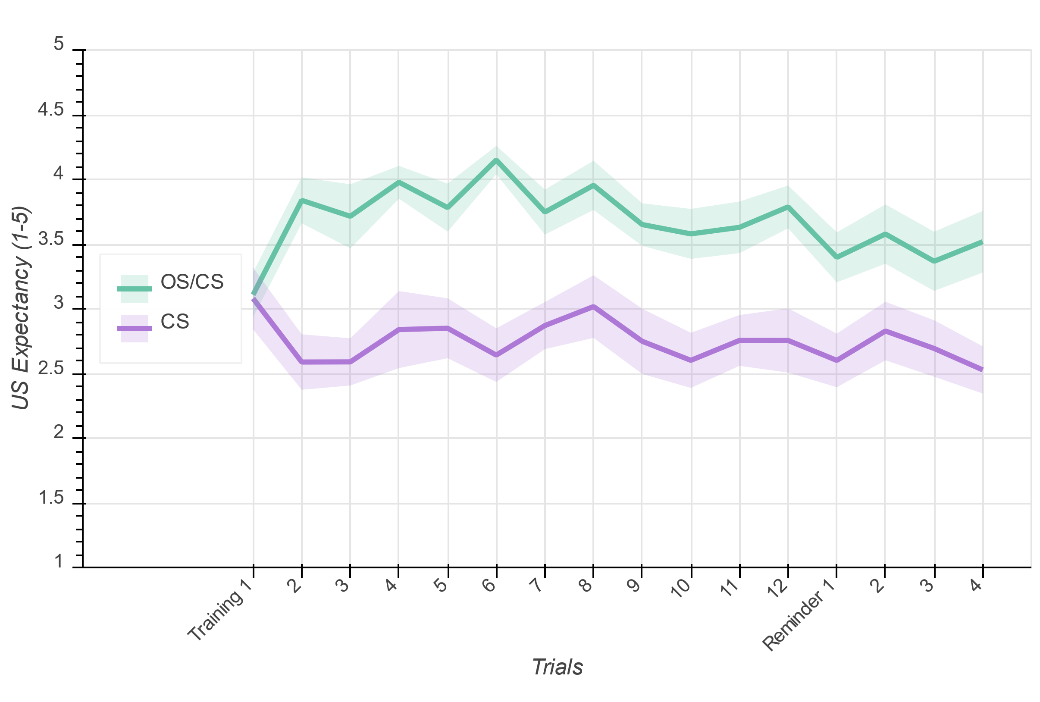


**Fig A. Supplementary Experiment Training Results.** Figure shows that participants had significantly greater US expectancy for the 75%-reinforced OS/CS trials than the 25%-reinforced CS trials for all trial except trial 1. “OS/CS” refers to trials in which the occasion setter and CS were both presented; “CS” refers to CS-alone trials. Results are collapsed across both OS and CS stimulus sets.

Furthermore, for the computational modeling, we multiplied the Δ formulas for the 1^st^-order positive occasion setting variables (P and N) with free leaky memory parameters (ί; “iota”) for each occasion setter, allowing them to leak their P and N values. We compared two 1^st^-order occasion setting models that either included or excluded the ί parameters using the WAIC as an estimate of model fit. The results showed that the leaky memory model including ί parameters had a better fit than the non-leaky memory model excluding the ί parameters (WAIC: ί model = 2830; non-ί model = 2946, respectively; lower is better). To help visualize the impact of the leaky memory parameters on our model’s ability to predict partial reinforcement, Fig C shows examples of two participants’ data from each model, where the orange line indicates their actual US expectancy, and the blue line indicates model-predicted US expectancy. Despite the relatively inconsistent nature of reinforcement, the model was able to track participants’ US expectancy quite well. Additionally, we would expect ί estimates for OS/CS trials to be near .75 (i.e., 75% reinforcement; ί_P indicates the leak from OS/CS trials) and near .25 for CS trials (i.e., 25% reinforcement; ί_N indicates the leak from CS-only trials). This occurred with our example two participants: Participant 1 (ί_P = .743, ί_N = .155, α = .854) and Participant 2 (ί_P = .726, ί_N = .201, α = .877). Functionally, the ί values inform us of the participants’ perceived reinforcement rate of each trial type. Overall, our computational model is able to predict occasion setting partial reinforcement rates accurately, and including the ί parameters improves model fit beyond excluding them.

With Leaky Memory ί Parameters

No Leaky Memory ί Parameters

Participant 1

Participant 2

**Fig B. Supplementary Experiment Real vs Model-Predicted Responding.** Two example participants’ modeling results are provided from the experiment in which the orange line indicates real US expectancy data, and the blue line indicates model-predicted US expectancy. Left column shows our 1^st^-order occasion setting model using ί leaky memory parameters with the two participants; right column shows the same two participants with a model that excludes the leaky memory parameters (but is otherwise the same as the leaky memory model). Model fit results showed that the leaky memory model was a better fit than the non-leaky memory model (WAIC = 2830 vs 2946, respectively). To illustrate this, the example participant results in this figure show that the model-predicted data is a stronger match with the real data when including the leaky memory parameters (left column) vs excluding them (right column). Note that the left column’s model predictions generally hover around |.5|, which is what would be expected with a 75% / 25% reinforcement rate when put on a -1 to 1 scale.


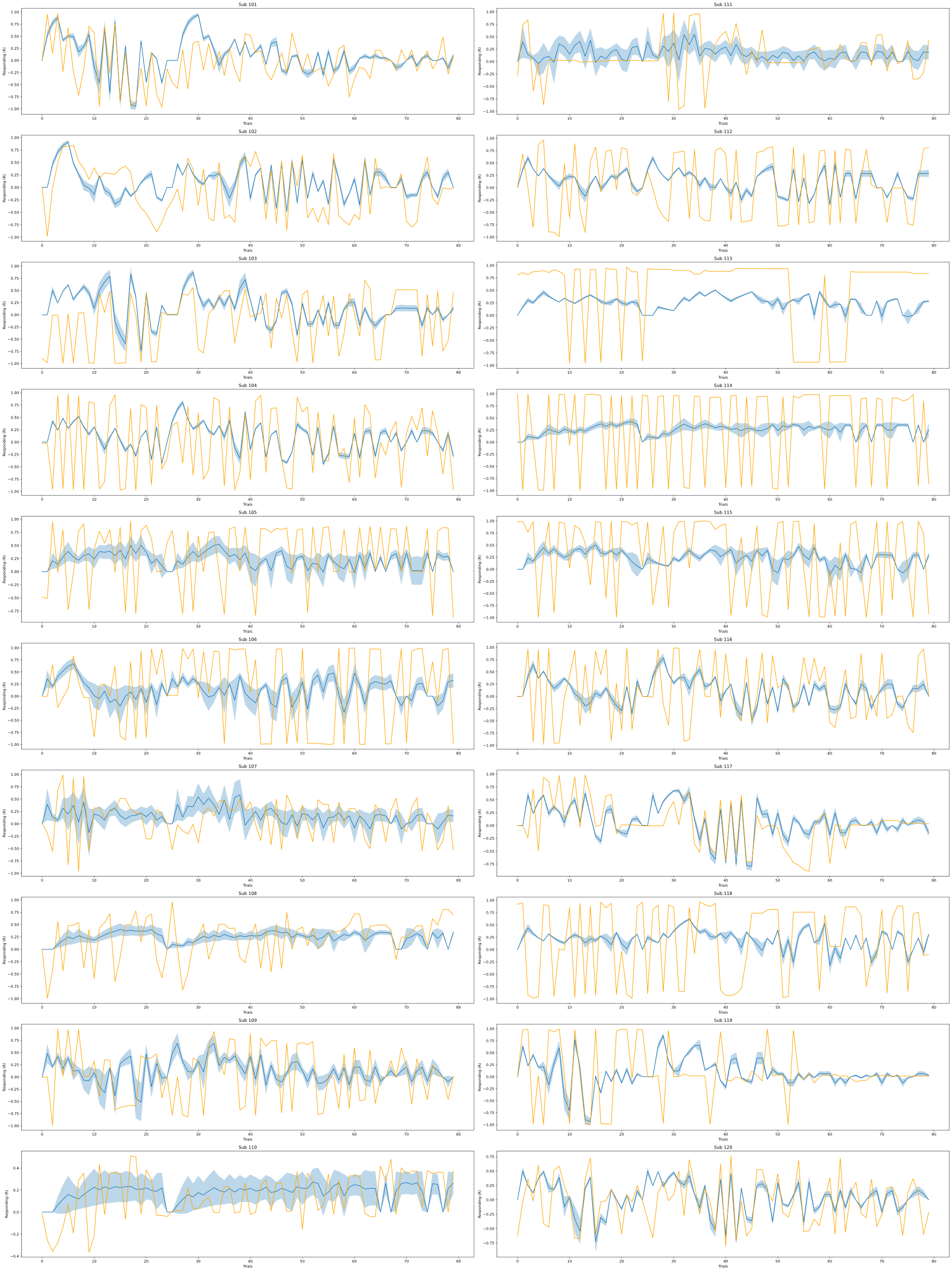

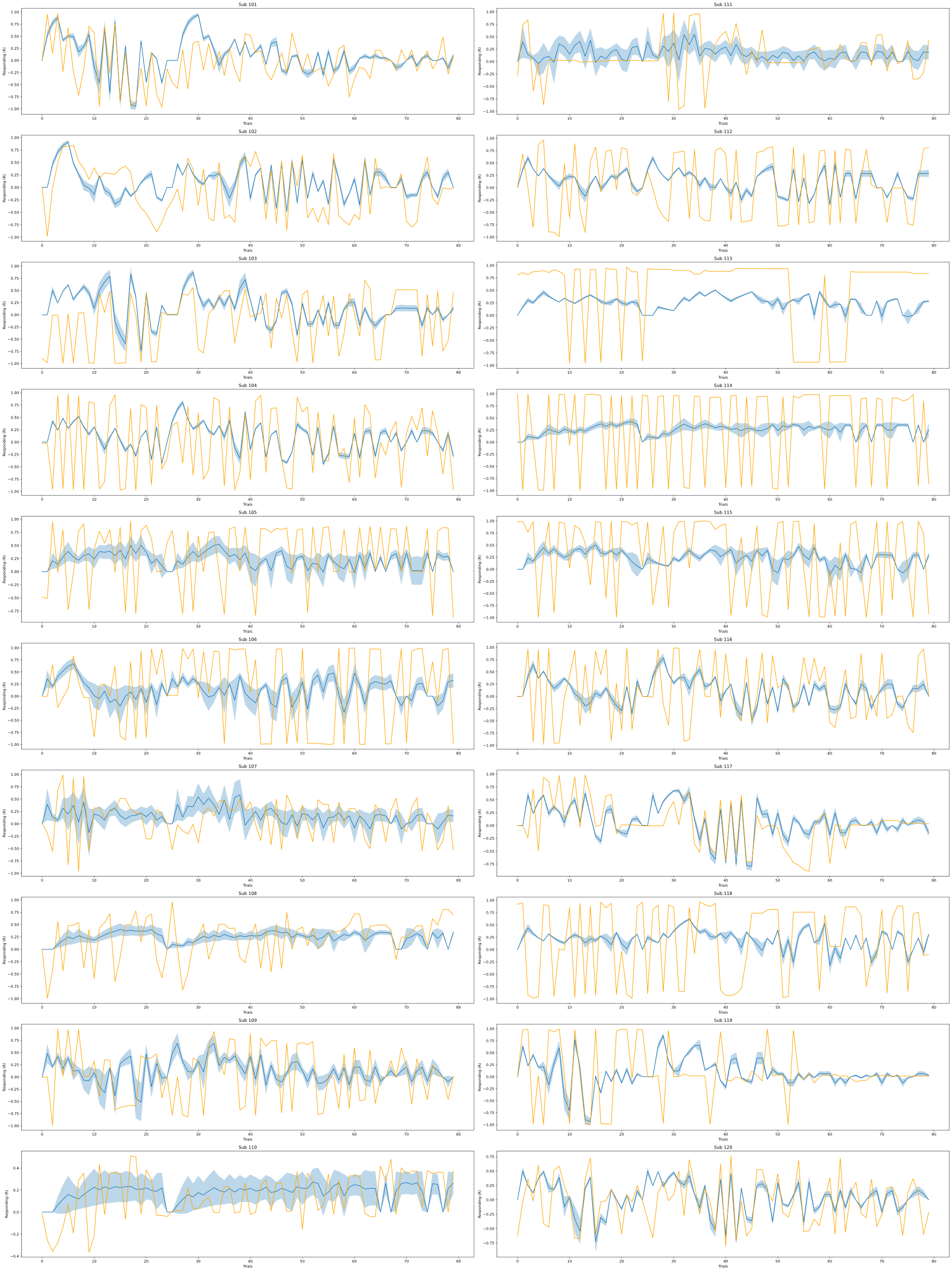

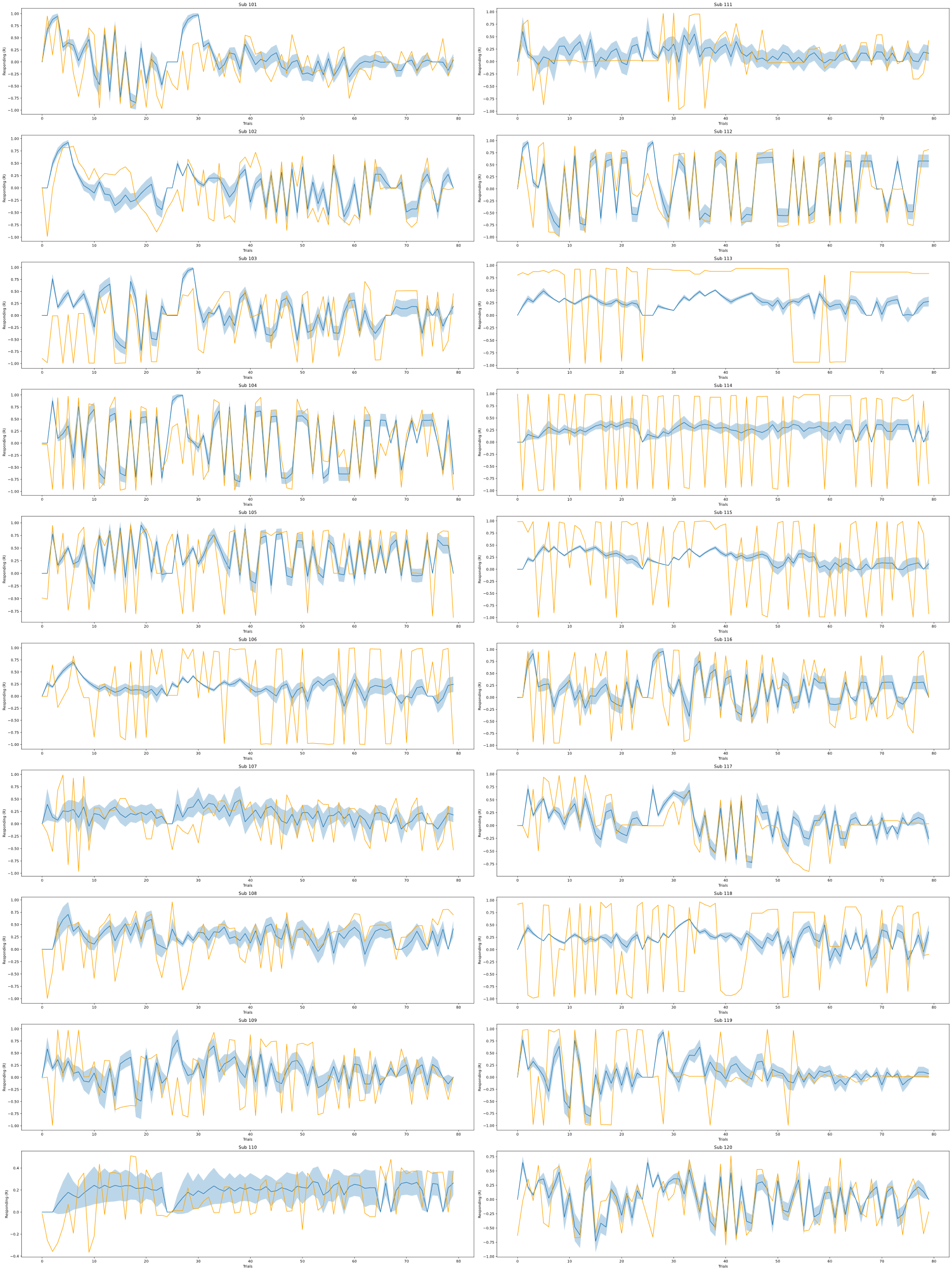

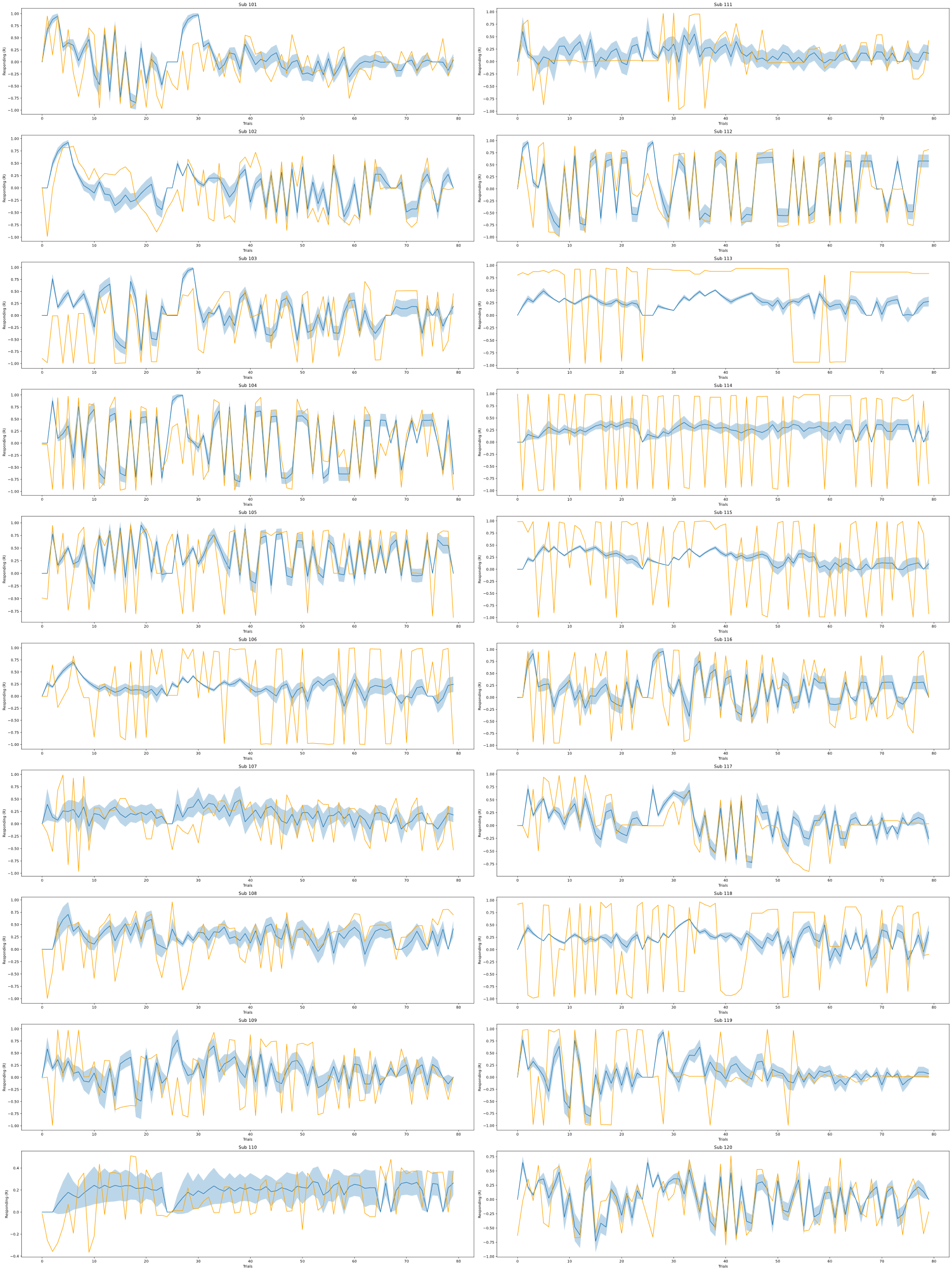

Supplement: S8 Text — Contains two figures: i) Fig A. Supplementary Experiment Training Results, and ii) Fig B. Supplementary Experiment Real vs Model-Predicted Responding. (DOCX) [file pcbi.1010410.s009.docx]
