# Supplementary material for: Ambiguity drives higher-order Pavlovian learning
Source: PLoS Comput Biol. 2022 Sep 9;18(9):e1010410. doi: 10.1371/journal.pcbi.1010410 (PMC9491594; doi:10.1371/journal.pcbi.1010410)
Supplement: S9 Text — (DOCX) [file pcbi.1010410.s010.docx]

**S9:** *Additional Factors That Could Affect Whether Occasion Setting Is Learned*

Our model incorporates many known aspects of occasion setting. However, there are additional factors that could affect whether occasion setting is learned. First, in our experiments, all stimuli were sufficiently salient to be detected without much effort. However, in the real world, detecting and learning about occasion setters likely requires more effort, and we might not even know if occasion setters even exist for a given CS (e.g., it could instead be simple partial reinforcement). This uncertainty could dissuade individuals from putting in the effort to search for or learn about potential occasion setters. Second, time pressure could also dissuade individuals from searching for occasion setters. For example, perhaps an individual has noticed that the taste of a specific brand and type of coffee is highly variable. Under time constraints at the grocery store, the individual might not try to *learn* what disambiguates whether the coffee will taste good or not; however, if the individual has already learned this coffee’s quality depends on which geographical region a given batch was grown in, they may quickly *detect* that occasion setter (i.e., read where the coffee came from on the bag) – even under time constraints. Third, financial (or other) costs of finding occasion setters could be worthwhile or a deterrent. This may depend on the probability of reinforcement with or without the occasion setter. For example, if OS/CS vs CS trials have reinforcement rates of 100%/0% vs 60%/40%, it would be more worthwhile to determine if an occasion setter is present if it predicts 100% (vs 0%) reinforcement rather than 60% (vs 40%). Similarly, the utility of finding an occasion setter could be affected by the probability that a CS will be presented alone vs with an occasion setter. For example, if 90% of CS trials are the CS alone and 10% of trials are OS/CS, it would be less worthwhile to incur the costs of finding out if an occasion setter is present (since the odds that it is present are low) as compared to 50% of trials being OS/CS and 50% being CS alone. Fourth, an additional constraint-based question is how many hierarchies people are capable of learning and how many hierarchies people are willing to learn in real life. The “recipe” underlying our formulas can be expanded to include an infinite number of hierarchies, including 3^rd^-order occasion setting and 4^th^-order occasion setting. However, there comes a point at which the number of hierarchies is too complicated for humans to learn in an experiment and too complicated to learn or implement in real-life situations. Based on our experiment, people are capable of learning 2^nd^-order occasion setting, but it remains untested whether this occurs in real-life situations or whether people are able to experimentally learn 3^rd^ or 4^th^-order occasion setting. Relatedly, while an *n*th-level hierarchy might provide more accuracy than an (*n-1)*-level hierarchy, it might not be worth the costs of learning those advanced hierarchies due to the complexity. Computationally, there could come a point where WAIC scores favor a simpler but less accurate model (e.g., 3^rd^-order) compared to a more accurate but more complex model (e.g., 4^th^-order). The factors described above may be useful to investigate experimentally and potentially incorporate into future versions of the model.
